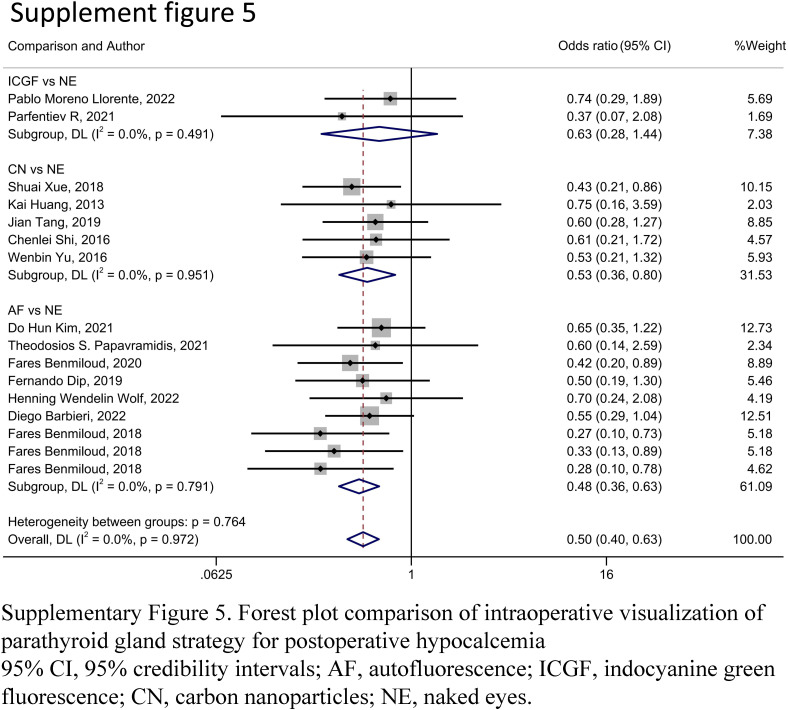# Intraoperative strategies in identification and functional protection of parathyroid glands for patients with thyroidectomy: a systematic review and network meta-analysis

**DOI:** 10.1097/JS9.0000000000000991

**Published:** 2023-12-11

**Authors:** Dengwei Lu, Bin Pan, Enjie Tang, Supeng Yin, Yiceng Sun, Yuquan Yuan, Tingjie Yin, Zeyu Yang, Fan Zhang

**Affiliations:** a Department of Breast and Thyroid Surgery, Chongqing General Hospital, Chongqing, China; b Thyroid, Breast and Vascular Surgery, Chongqing University FuLing Hospital, Chongqing, China; c Graduate School of Medicine, Chongqing Medical University, Chongqing, China; d Epidemiology Department, College of Preventive Medicine, Army Medical University (Third Military Medical University), Chongqing, China

**Keywords:** Autofluorescence, carbon nanoparticles, indocyanine green fluorescence, parathyroid gland, thyroid surgery

## Abstract

**Background:**

This study aimed to assess the benefits and limitations of four intraoperative visualization of parathyroid gland (IVPG) strategies in the identification and functional protection of parathyroid glands (PGs).

**Methods:**

We searched PubMed, the Cochrane Central Register of Controlled Trials, CNKI, EMBASE, Web of Science and Google Scholar databases until 30 June 2023. Four IVPG strategies were composed of the naked eyes (NE) and three imaging strategies: autofluorescence (AF), indocyanine green fluorescence (ICGF), and carbon nanoparticles (CN). We performed a pairwise meta-analysis (PMA) for direct comparisons and a Bayesian network meta-analysis (NMA) for indirect comparisons.

**Results:**

A total of 29 eligible studies were included. According to NMA and PMA, AF had significantly lower rates of postoperative hypocalcemia and hypoparathyroidism, PG inadvertent resection, and PG auto-transplantation compared to NE, while had significantly higher rate of PG identification. CN showed significantly lower rates of postoperative hypocalcemia and hypoparathyroidism, and PG inadvertent resection compared to NE in PMA and NMA. ICGF showed a significantly higher rate of PG auto-transplantation compared to NE in PMA and AF in NMA. According to SUCRA values, AF showed the best advantage in reducing the rate of postoperative hypocalcemia (0.85) and PG inadvertent resection (0.89), and increasing the rate of PG identification (0.80). CN had the greatest advantage in reducing the rate of postoperative hypoparathyroidism (0.95). ICGF ranked the highest in the rate of PG auto-transplantation (0.98).

**Conclusions:**

Three imaging strategies demonstrate significant superiority over NE in the intraoperative PG identification and functional protection. AF is the best strategy in reducing the incidence of postoperative hypocalcemia, increasing the rate of PG identification, and reducing the rate of PG inadvertent resection and auto-transplantation. ICGF has great value in assessing PG viability, leading to the trend towards PG auto-transplantation. CN is the best strategy in reducing the incidence of postoperative hypoparathyroidism.

## Introduction

Highlights

- This is the first systematic review and network meta-analysis that provides a comprehensive comparison of four intraoperative visualization strategy of parathyroid gland (IVPG) strategies on the identification and preservation of parathyroid glands (PGs), and their functional protection.
- The three imaging strategies were significantly superior to naked eyes in identifying and preserving PGs, and protecting their function during surgery.
- Autofluorescence ranked the highest among four IVPG strategies in reducing the rate of postoperative hypocalcemia, increasing the rate of PG identification, and reducing the rates of PG inadvertent resection and auto-transplantation.
- Carbon nanoparticles had the best superiority among four IVPG strategies in reducing the rate of postoperative hypoparathyroidism.
- ICGF showed the best performance among four IVPG strategies in increasing the rate of PG auto-transplantation.

Postoperative hypocalcemia is an obstinate complication of thyroidectomy, directly associated with the inadvertent resection of parathyroid glands (PGs) or the devascularization of their vessels during thyroid surgery^1^. The incidence of transient hypocalcemia ranges from 3.15 to 64.25%, while permanent hypocalcemia occurs in 0 to 6.84% of cases^2^. Patients with hypocalcemia experience a series of symptoms, including peripheral paraesthesia, muscle cramps, tetany, and even mortality, who need transient or lifelong calcium and vitamin D supplementation^3,4^, leading to the concern of intraoperative functional protection of PGs. Importantly, accurate identifying PGs is essential prerequisite for intraoperative protection of PGs.

Conventional intraoperative visualization strategy of parathyroid glands (IVPG) relies on the naked eye (NE), which is subjective and usually influenced by the experiences of surgeons. Therefore, developing objective techniques to achieve the IVPG for protecting PGs intraoperatively is of importance^5^. Recently, some imaging strategies, such as autofluorescence (AF), indocyanine green fluorescence (ICGF), and carbon nanoparticles (CN), have shown a promising potential in enhancing PG identification and reducing the incidence of postoperative hypocalcemia when compared to NE^6–8^. PGs exhibit remarkably stronger autofluorescence than surrounding tissues when exposed to near-infrared light, enabling their identification during surgery^9^. Indocyanine green (ICG), as a near-infrared fluorescing exogenous agent, rapidly binds to plasma proteins and generates fluorescence upon intravenous injection, which can contribute to identifying PGs and protecting their function^10^. Carbon nanoparticles can blacken the thyroid gland, while PGs remain unstained, facilitating their identification^6^. However, each imaging strategy has its respective benefits and limitations in clinical applications, which have yet to be compared.

This study aimed to comprehensively analyze the efficacy of four IVPG strategies in the identification and preservation of PGs, and their functional protection. Due to the deficiency of relevant studies, direct two-by-two comparisons of imaging strategies were not feasible. To address this limitation, network meta-analyses (NMA) were employed to synthesize both direct and indirect evidence from a network of trials that compared multiple interventions, which enables the comparisons between all three imaging strategies and NE, as well as among the different imaging strategies themselves. Notably, we conducted ranking analyses of the four IVPG strategies to illustrate their respective benefits and limitations.

## Materials and methods

### Meta-analysis

This network meta-analysis was conformed with PRISMA (Preferred Reporting Items for Systematic Reviews and Meta-Analyses), Supplemental Digital Content 1, http://links.lww.com/JS9/B530, Supplemental Digital Content 2, http://links.lww.com/JS9/B531 and AMSTAR (Assessing the methodological quality of systematic reviews) Guidelines^11–13^, Supplemental Digital Content 3, http://links.lww.com/JS9/B532.

### Literature search

We performed a systematic literature search in PubMed, the Cochrane Central Register of Controlled Trials, EMBASE, Web of Science, CNKI, and Google Scholar databases up to June 30, 2023. The keywords were (“thyroid surgery” OR “thyroid operation” OR “thyroidectomy”) AND (“indocyanine green” OR “near-infrared fluorescence” OR “autofluorescence” OR “carbon nanoparticles”). Only reports written in English were reviewed, and the study type was either an randomized controlled trial (RCT) or NRS.

Two investigators independently conducted the literature search, and disagreements were resolved by consensus. Abstracts of the retrieved studies were reviewed and excluded if deemed irrelevant. The full text was checked to determine the final eligible articles. Discrepancies were resolved through discussion with a third investigator. The reference lists of the selected articles were reviewed to ensure that more relevant studies were obtained. The references were managed using the EndNote X9 software.

### Study design

Studies were considered eligible based on the following inclusion criteria: (1) thyroidectomy due to benign or malignant thyroid disease; (2) a prospective or retrospective control study protocol; (3) AF, ICGF, or CN used as an IVPG strategy; and (4) the incidence of postoperative hypocalcemia, the rate of postoperative hypoparathyroidism, the rate of intraoperative PG identification, auto-transplantation and inadvertent resection were defined as outcomes of these studies. Studies were excluded based on the following criteria: (1) conference abstracts, reviews, case reports, animal experiments, commentaries, discussions, letters, and non-control studies; (2) parathyroidectomy with thyroidectomy; (3) incomplete key outcomes of interest.

### Data extraction

Two investigators independently extracted the following data from each study: (1) characteristics of the study including the first author, year and country of publication, study design, IVPG strategy employed, gender composition, operation method, and postoperative thyroid pathology; (2) primary outcomes, including the proportion of patients with transient hypocalcemia (defined as calcium levels less than 8.0 mg/dl or corrected calcium less than 2.00 mmol/l within 6 months after surgery) and the incidence of postoperative hypoparathyroidism (These indicators were from patients who underwent total thyroidectomy); (3) secondary outcomes included the rate of PG identification, inadvertent resection, and auto-transplantation (These indicators were from patients who underwent who underwent unilateral and total thyroidectomy).

### Quality assessment

RCTs and NRSs were classified according to the study design; the latter included prospective cohort and case-control studies. Two investigators independently evaluated the quality of the included studies using the Cochrane Collaboration tool for RCTs and the Newcastle-Ottawa Scale (NOS) for NRSs in line with the suggestions of the Cochrane Collaboration. Differences were estimated by a third investigator or team consensus.

### Statistical analysis

The interventions were categorized into four groups: AF, ICGF, CN, and NE. We performed a pairwise meta-analysis (PMA) for direct comparisons using Stata 12.0. Bayesian network meta-analysis (NMA) for indirect comparisons by RStudio (version 4.2.1) with the “BUGSnet” package^14^. The outputs for categorical variables are odds ratios (OR) with corresponding 95% CI. Markov chain Monte Carlo simulations were performed to fit the random effect models. The analysis was performed using 1000 burn-ins, 50 000 iterations, and 20 000 adaptations, after which the model fit was tested using a leverage diagram. The consistency between direct and indirect comparisons was tested, and a random-effects model was directly selected using the deviance information criterion (DIC)^14^. The loop-specific approach was used to evaluate the agreement between direct and indirect effects in all closed loops^15–17^. *P* value greater than or equal to 0.05 suggested that the consistency of the model was satisfactory. We used the surface under the cumulative ranking curve (SUCRA) to rank different IVPG strategies for each outcome. Pairwise comparisons of different comparisons are recommended for OR with proportional CI. Funnel plots were utilized in Stata 12.0 to estimate the publication bias of each observational outcome.

## Results

### Literature search

**Figure 1:**
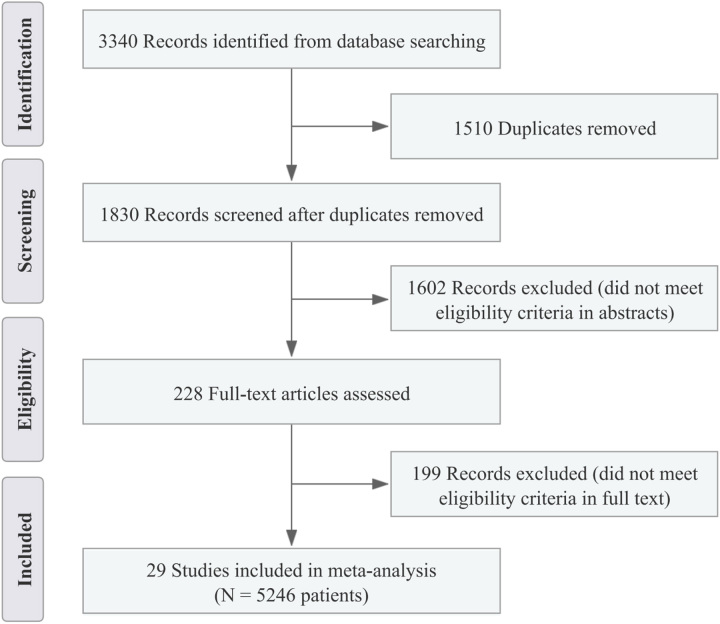
PRISMA flow diagram of study identification and selection.

Following the previous search strategy, a total of 3340 potentially relevant articles were identified (Supplementary Table 1, Supplemental Digital Content 4, http://links.lww.com/JS9/B533), and 228 remained after removing duplicates and initial screening. Then, a full-text review was conducted to exclude articles not meeting the inclusion criteria, and 29 studies included 5246 patients were identified and enroled in the meta-analysis^18–46^. The PRISMA flow diagram shows the details of the article selection and exclusion procedure (Fig. 1).

### Study characteristics

The characteristics of the included studies are summarized in Supplementary Table 2, Supplemental Digital Content 4, http://links.lww.com/JS9/B533. The demographic and clinical characteristics of the included patients showed that the mean age of the patients was 45.6 ± 11.3 years and a higher proportion of females (77.82%) than males. Among the 29 studies, 8 studies were RCTs and 21 studies were NRSs. All of studies compared the imaging strategies (AF, ICGF, and CN) with NE.

**Figure 2:**
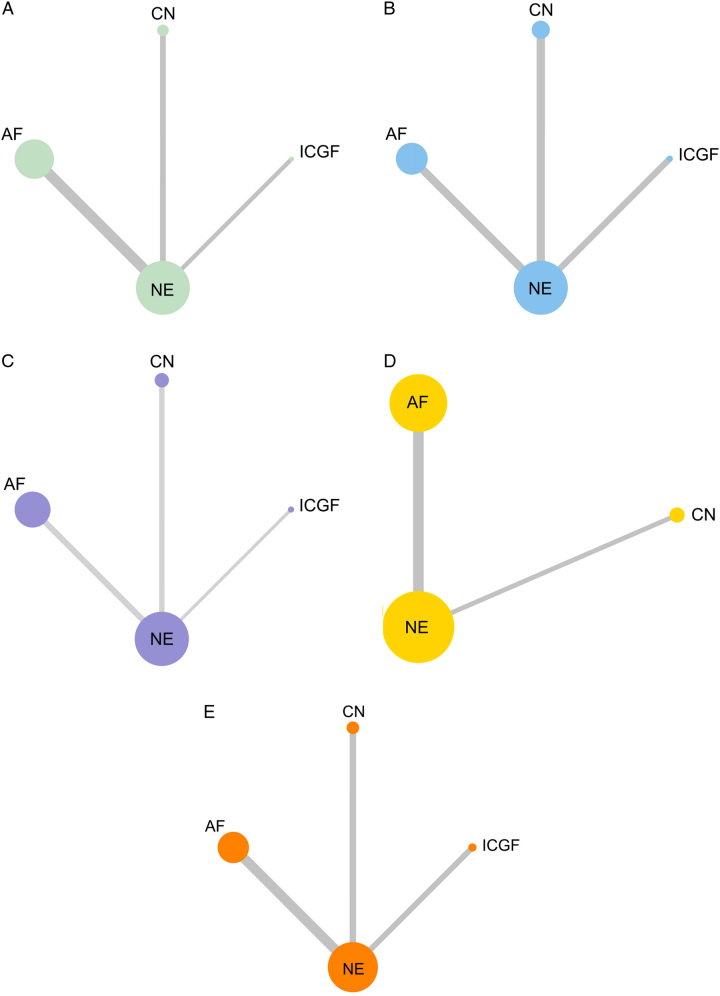
Network relationship plots of four IVPG strategies for five observational outcomes. (A) Incidence of postoperative hypocalcemia; (B) Incidence of postoperative hypoparathyroidism; (C) Rate of PG identification; (D) Rate of PG inadvertent resection; (E) Rate of PG auto-transplantation. The area of each circle represents the number of patients included and the thickness of the lines linking the two IVPG strategies represents the number of articles. AF, autofluorescence; CN, carbon nanoparticles; ICGF, indocyanine green fluorescence; IVPG, intraoperative visualization of parathyroid gland; NE, naked eyes; PG, parathyroid gland.

The network relationships between different IVPG strategies are shown in Fig. 2. The results of the bias risk assessment were delineated in Supplementary Table 3, Supplemental Digital Content 4, http://links.lww.com/JS9/B533 and Supplementary Table 4, Supplemental Digital Content 4, http://links.lww.com/JS9/B533. No study that scored lower than 6 on the NOS were regarded as of low quality after assessment. One RCT had a high risk of bias according to the Cochrane Collaboration tool. A quantitative synthesis of the evidence through a network meta-analysis was deemed appropriate, given the comparability in study design, outcome measures, patients involved, and inclusion and exclusion criteria.

**Figure 3:**
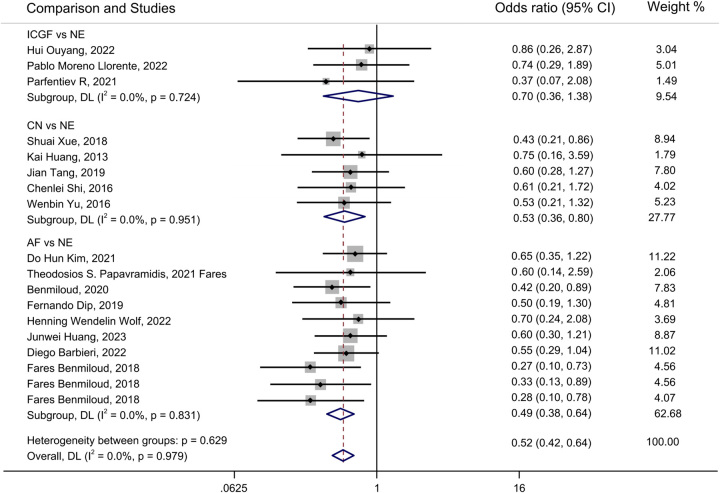
Forest plot comparison of intraoperative visualization of parathyroid gland strategy for postoperative hypocalcemia. AF, autofluorescence; CN, carbon nanoparticles; ICGF, indocyanine green fluorescence; NE, naked eyes.

**Figure 4:**
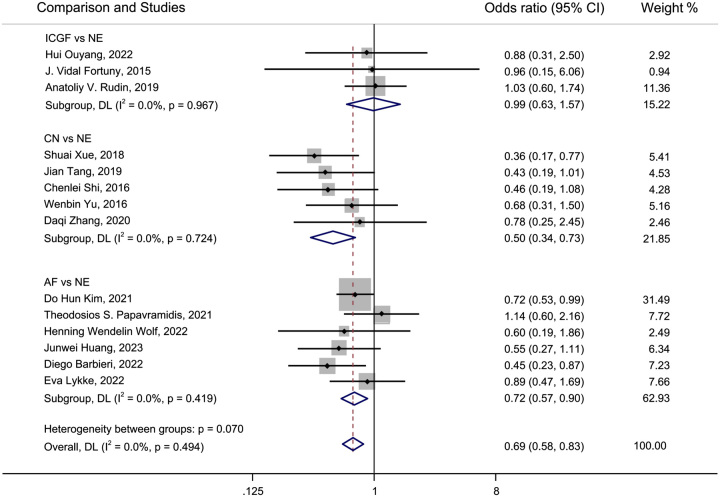
Forest plot comparison of intraoperative visualization of parathyroid gland strategy for postoperative hypoparathyroidism. AF, autofluorescence; CN, carbon nanoparticles; ICGF, indocyanine green fluorescence; NE, naked eyes.

**Figure 5:**
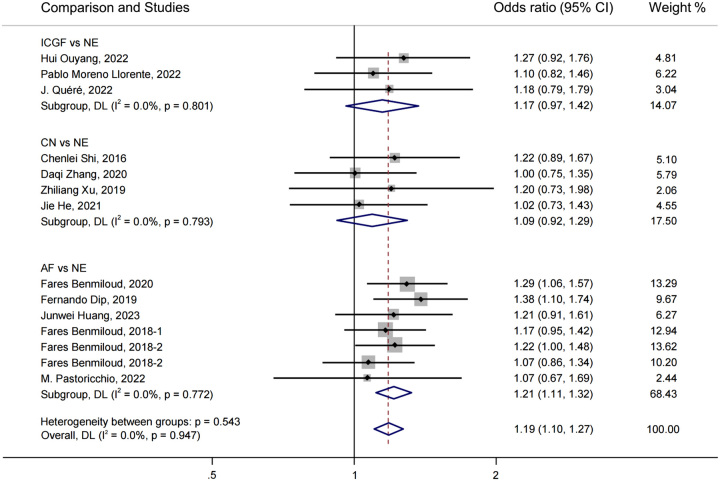
Forest plot comparison of intraoperative visualization of parathyroid gland strategy for rate of parathyroid gland identification. AF, autofluorescence; CN, carbon nanoparticles; ICGF, indocyanine green fluorescence; NE, naked eyes.

**Figure 6:**
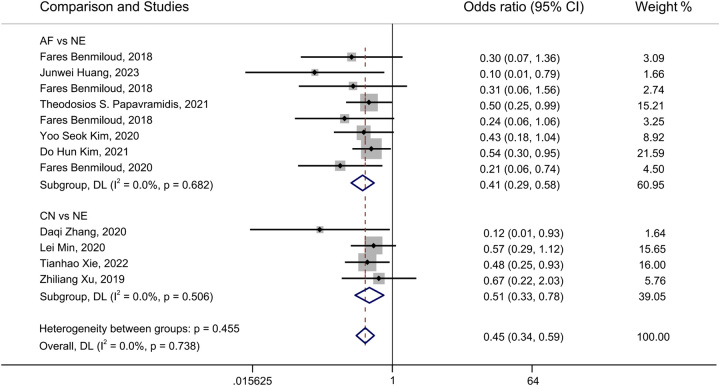
Forest plot comparison of intraoperative visualization of parathyroid gland strategy for rate of parathyroid gland inadvertent resection. AF, autofluorescence; CN, carbon nanoparticles; ICGF, indocyanine green fluorescence; NE, naked eyes.

**Figure 7:**
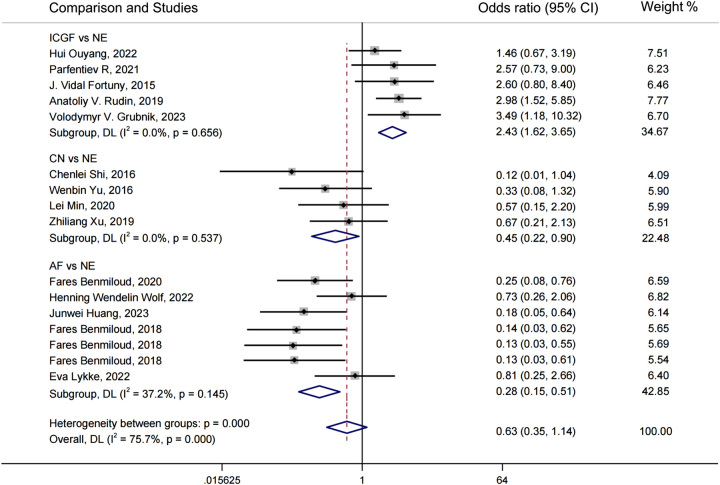
Forest plot comparison of intraoperative visualization of parathyroid gland strategy for rate of parathyroid gland auto-transplantation. The Heterogeneity between groups (I^2^=75.7%, *P*<0.0001) has no practical significance, and there is no Heterogeneity between studies in each group. AF, autofluorescence; CN, carbon nanoparticles; ICGF, indocyanine green fluorescence; NE, naked eyes.

**Figure 8:**
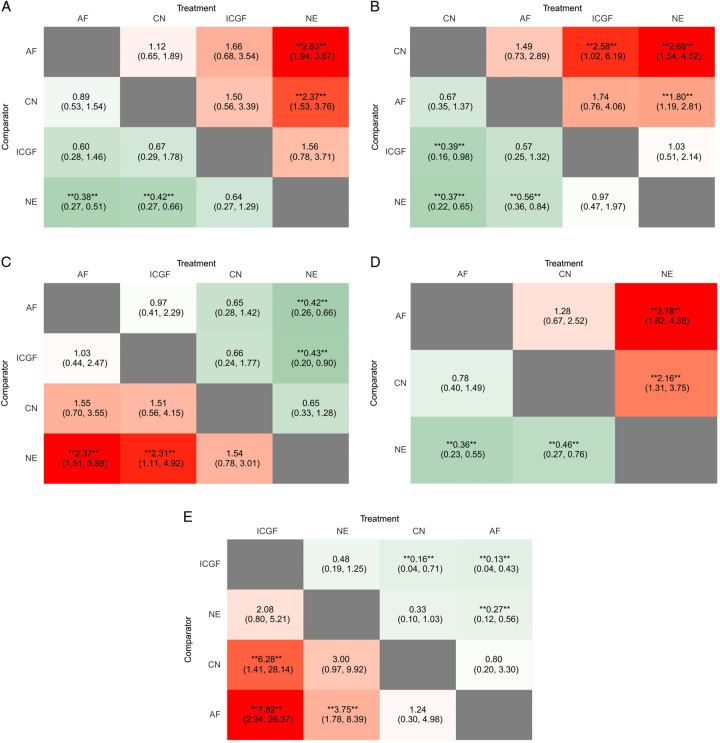
Heat plots of league table of four IVPG strategies for five observational outcomes. (A) Incidence of postoperative hypocalcemia; (B) Incidence of postoperative hypoparathyroidism; (C) Rate of PG identification; (D) Rate of PG inadvertent resection; (E) Rate of PG auto-transplantation. AF, autofluorescence; CN, carbon nanoparticles; ICGF, indocyanine green fluorescence; IVPG, intraoperative visualization of parathyroid gland; NE, naked eyes; PG, parathyroid gland.

A forest plot of pairwise IVPG strategies for different observational outcomes is displayed in Figs. 3 – 7. I^2^ less than 50% indicated satisfactory homogeneity. The heat plots of league table of the four IVPG strategies for different observational outcomes is shown in Fig. 8. After 50 000 iterations, the model achieved ideal convergence (Supplementary Figure 1, Supplemental Digital Content 5, http://links.lww.com/JS9/B534). The funnel plot showed a low possibility of publication bias in the different IVPG strategies (Supplementary Figure 2, Supplemental Digital Content 6, http://links.lww.com/JS9/B535). The cumulative ranking plots and rank nomograms of the four IVPG strategies are shown in Supplementary Figure 3, Supplemental Digital Content 7, http://links.lww.com/JS9/B536.

### Primary outcome

#### Postoperative hypocalcemia

Sixteen studies comprising 3142 patients reported postoperative hypocalcemia^18–20,25–29,35–42^. By analyzing these studies, we found that AF had a significantly lower incidence of postoperative hypocalcemia than NE in PMA (OR: 0.49, 95% CI: 0.38, 0.64) and NMA (OR: 0.38, 95% CI: 0.27, 0.51) (Figs. 3, 8A). CN had a significantly lower incidence of postoperative hypocalcemia than NE in PMA (OR: 0.53, 95% CI: 0.36, 0.80) and NMA (OR: 0.42, 95% CI: 0.27, 0.66) (Figs. 3, 8A). The comparison of the incidence of postoperative hypocalcemia between ICGF and NE showed no statistical difference in PMA (OR: 0.70, 95% CI: 0.36, 1.38) or NMA (OR: 0.64, 95% CI: 0.27, 1.29) (Figs. 3, 8A). According to SUCRA values, AF had the best advantage in reducing the rate of postoperative hypocalcemia (0.85), followed by CN (0.71), ICGF (0.41), and NE (0.03) (Supplementary Table 5, Supplemental Digital Content 4, http://links.lww.com/JS9/B533).

#### Postoperative hypoparathyroidism

Fourteen studies comprising 2315 patients reported postoperative hypoparathyroidism^18,21,22,25,27–30,35,36,39–41,45^. AF had a significantly lower incidence of postoperative hypoparathyroidism than NE in PMA (OR: 72, 95% CI: 0.57, 0.90) and NMA (OR: 0.56, 95% CI: 0.36, 0.84) (Figs. 4, 8B). CN had a significantly lower incidence of postoperative hypocalcemia than NE in PMA (OR: 0.50, 95% CI: 0.34, 0.73) and NMA (OR: 0.37, 95% CI: 0.22, 0.65) (Figs. 4, 8B). The comparison of the incidence of postoperative hypoparathyroidism between ICGF and NE showed no statistical difference in PMA (OR: 0.99, 95% CI: 0.63, 1.57) or NMA (OR: 0.97, 95% CI: 0.47, 1.97) (Figs. 4, 8B). According to SUCRA values, CN had the best advantage in reducing the rate of postoperative hypoparathyroidism (0.95), followed by AF (0.67), ICGF (0.22), and NE (0.16) (Supplementary Table 5, Supplemental Digital Content 4, http://links.lww.com/JS9/B533).

### Secondary outcomes

#### Rate of PG identification

Twelve with 1,723 patients containing 7,271 parathyroid glands were analyzed^18,19,23,28,30,32,33,37,38,40,42,46^. AF had a significantly higher rate of PG identification than NE in PMA (OR: 1.21, 95% CI: 1.11, 1.32) and NMA (OR: 2.37, 95% CI: 1.51, 3.88) (Figs. 5, **8 C** ). ICGF had a significantly higher rate of PG identification than NE in NMA (OR: 2.31, 95% CI: 1.11, 4.92) (Fig. 8C), while in PMA, there was no significant difference (Fig. 5). There were no statistical differences in the comparison between CN and NE in PMA and NMA (Figs. 5, 8C). According to the SUCRA values, AF ranked the highest in increasing the rate of PG identification (0.80), ICGF ranked second (0.76), CN ranked third (0.41), NE ranked the lowest (0.04) (Supplementary Table 5, Supplemental Digital Content 4, http://links.lww.com/JS9/B533).

#### Rate of PGs inadvertent resection

Nine studies with 2747 patients containing 10 706 identified PGs were assessed^30,31,33,34,36,37,40,42,43^. As the lack of ICGF raw data, only three IVPG strategies (AF, CN, NE) were compared. AF had a significantly lower rate of PG inadvertent resection than NE in PMA (OR: 0.41, 95% CI: 0.29, 0.58) and NMA (OR: 0.36, 95% CI: 0.23, 0.55) (Figs. 6, 8D). CN had a significantly lower rate of PG inadvertent resection than NE in PMA (OR: 0.51, 95% CI: 0.33, 0.78) and NMA (OR: 0.46, 95% CI: 0.27, 0.76) ( **Figs. 6**, **8 D** ). However, there was no statistical difference between CN and AF in NMA (OR: 1.28, 95% CI: 0.67, 2.52) (Figs. 6, 8D). According to SUCRA values, AF had the best advantage (0.89) in reducing the rate of PG inadvertent resection, CN ranked second (0.61), and NE had the least advantage (0) (Supplementary Table 5, Supplemental Digital Content 4, http://links.lww.com/JS9/B533).

#### Rate of PG auto-transplantation

Thirteen studies with 2333 patients were included to assess the rate of PG auto-transplantation^18,20–22,24,28,29,31,33,39,40,42,45^. AF had a significantly lower rate of PG auto-transplantation than NE in PMA (OR: 0.28, 95% CI: 0.15, 0.51) and NMA (OR: 0.27, 95% CI: 0.12, 0.56) (Figs. 7, 8 E). The rate of PG auto-transplantation of ICGF was significantly higher than that of NE in PMA (OR: 2.43, 95% CI: 1.62, 3.65), while there was no statistical difference in NMA (OR: 2.08, 95% CI: 0.80, 5.21) (Figs. 7, 8E). ICGF had a significantly higher rate of PG auto-transplantation than AF (OR: 7.82, 95% CI: 2.34, 26.37) and CN (OR: 6.28, 95% CI: 1.41, 28.14) in NMA (Fig. 8E). CN had significant lower rate of PG auto-transplantation than NE in NMA (OR: 0.45, 95% CI: 0.22, 0.90), while there was no statistical difference between them in NMA (OR: 0.33, 95% CI: 0.10, 1.03) (Figs. 7, 8E). According to the SUCRA values, ICGF had the highest rate of PG auto-transplantation (0.98), followed by NE (0.68), CN (0.22), and AF (0.12) (Supplementary Table 5, Supplemental Digital Content 4, http://links.lww.com/JS9/B533).

### Subgroup analysis

Due to the surgical techniques may have certain effects on the outcomes, we conducted a subgroup analysis for open surgeries. Due to data limitations, we performed the subgroup analysis for open surgeries on the primary outcomes that included postoperative hypocalcemia and hypoparathyroidism. As shown in Supplementary Figures 4-7, Supplemental Digital Content 8, http://links.lww.com/JS9/B537, Supplemental Digital Content 9, http://links.lww.com/JS9/B538, Supplemental Digital Content 10, http://links.lww.com/JS9/B539, Supplemental Digital Content 11, http://links.lww.com/JS9/B540, AF had a significantly lower incidence of postoperative hypocalcemia and hypoparathyroidism than NE in PMA and NMA. CN had a significantly lower incidence of postoperative hypocalcemia and hypoparathyroidism than NE in PMA and NMA. The comparison of the incidence of postoperative hypocalcemia and hypoparathyroidism between ICGF and NE showed no statistical difference in PMA or NMA. According to SUCRA values, AF had the best advantage in reducing the rate of postoperative hypocalcemia, followed by CN, ICGF, and NE (Supplementary Table 6, Supplemental Digital Content 4, http://links.lww.com/JS9/B533). Regarding the rate of postoperative hypoparathyroidism, CN had the best advantage, followed by AF, ICGF, and NE (Supplementary Table 6, Supplemental Digital Content 4, http://links.lww.com/JS9/B533).

## Discussion

This is the first systematic review and network meta-analysis that provides a comprehensive comparison of four IVPG strategies on the identification and functional protection of PGs. We ranked the four IVPG strategies by SUCRA values to reveal their respective benefits and limitations. The present results confirmed that AF had the best advantage in reducing the incidence of postoperative hypocalcemia and the rate of PG inadvertent resection, and increasing the rate of PG identification. CN ranked the highest in reducing the incidence of postoperative hypoparathyroidism. ICGF showed the best performance in increasing the rate of PG auto-transplantation.

Parathyroid tissue contains fluorescent compounds with aromatic groups that emit infrared light at wavelength of 820–830 nm when excited by light at wavelength of 760–770 nm^9,47^. The fluorescence intensity of PGs is 2.4–8.5 times stronger than that of the surrounding tissues (such as the thyroid, fat, trachea, and muscle), which can be used to identify PGs^47^. Currently, a series of studies revealed the effectiveness of AF in identifying PGs during thyroid surgery^8,37–39^. This study confirmed that AF had significantly lower rates of postoperative hypocalcemia, postoperative hypoparathyroidism, PG inadvertent resection, and PG auto-transplantation, and significantly higher rate of PG identification when compared to NE. Among the four IVPG strategies, AF showed the best advantage in increasing the rate of PG identification, reducing the incidence of postoperative hypocalcemia, and reducing the rates of PG inadvertent resection and auto-transplantation. Theoretically, AF can assist surgeons in identifying more PGs before dissection, leading to a preservation of more PGs on-site and a reduction in inadvertent resection^48^. Consequently, the rates of PG auto-transplantation and postoperative hypocalcemia decrease, which is consistent with our results. Additionally, AF cannot assess the blood supply of PGs, surgeons may tend to preserve the identified PGs in situ, and therefore, the rate of PG auto-transplantation becomes lower^19^. These findings highlight the significant potential of AF in enhancing parathyroid protection during thyroid surgery^49^.

Indocyanine green is a near-infrared exogenous fluorescent agent that rapidly combines with plasma proteins after intravenous injection. The elicited fluorescence, when exposed to near-infrared light, contributes to the assessment of blood perfusion of PGs, which plays a crucial role in identifying PGs and predicting the incidence of postoperative hypocalcemia^10,50^. The results showed that, although ICGF can significantly increase the PG identification rate than NE, there were no significant differences in the incidence of postoperative hypocalcemia and hypoparathyroidism between them. This may because the ICGF had a significantly higher rate of PG auto-transplantation than NE. Although more higher PGs were identified by ICGF during the surgery, the blood perfusion of these PGs can be assessed by ICGF. The PG lacking ICG fluorescence intensity may be perceived as having impaired blood supply, leading to a trend towards parathyroid auto-transplantation^51^. We found that although ICGF was superior to NE in identifying PGs and reducing the incidence of postoperative hypocalcemia, it was inferior to AF in this aspect. This is attributed to the uptake of indocyanine green in the thyroid gland, which limits its ability to accurately identify PGs^52,53^. Therefore, the benefits and limitations of AF and ICGF in the identification and blood supply assessment of PGs are considered complementary. Based on these findings, our centre conducted a randomized controlled trial, revealing that the combined use of AF and ICGF could reduce the risk of transient postoperative hypocalcemia, enhance the ability to identify and preserve PGs, and improve the accuracy of PG perfusion evaluation during surgery^54^. However, due to the data deficiency of ICGF regarding the rate of PG inadvertent resection, we were unable to analyze the performance of ICGF in this aspect. This is because ICGF is typically used to assess the blood supply of PGs after thyroid resection and cannot identify isolated PGs^55^. Additionally, the high costs and cumbersome operation of AF or ICGF equipment pose critical limitations in their widespread application and promotion^55–57^. Therefore, it is worth developing a more convenient device that combines the capabilities of both techniques.

Carbon nanoparticle (CN) was applied in the form of a standard CN suspension injection (1 ml: 50 mg)^28,58^. The suspension does not enter the blood circulation and has no toxic side effects on the human body. The nanocarbon suspension comprises nanosized carbon particles with an average diameter of 150 nm. The cells gap between capillary endothelial cells is 20–50 nm, and the capillary lymphatic endothelial cell gap is 120–500 nm with a hypoplasia of the basement membrane. Thus, nanocarbon is unable to enter the blood vessels when it is injected into the thyroid tissue, and it will rapidly enter lymphatic vessels or the lymphatic capillaries through macrophage phagocytosis, and be retained in the lymph nodes. Furthermore, the thyroid and lymph in their drainage areas are stained in black in surgery. However, the parathyroid glands do not stain black, and thus, the black stained thyroid and lymph nodes can be identified and distinguished easily. Our results revealed that, compared to NE, CN significantly reduced the rate of PG inadvertent resection and the incidence of postoperative hypocalcemia and hypoparathyroidism. Among the three imaging strategies (AF, ICGF, CN), CN showed the least advantage in PG identification. This could be attributed to the potential difficulty in identifying PGs once the surgical field is stained due to improper injection^33,58^. Given the advantages and accessibility of CN, it can be applied in centres lacking intraoperative fluorescence imaging systems.

Although we performed a comprehensive comparison of the four IVPG strategies in identifying PGs and protecting their function, this study has several limitations. The surgical techniques may have certain effects on the outcomes. Therefore, we conducted a subgroup analysis for open surgeries and found that the results and conclusions remained unchanged, which means the conclusions of this study are reliable. However, we were unable to conduct subgroup analyses for all outcomes separately for different surgical methods due to the data deficiency. Additionally, due to the absence of the data of head-to-head control studies between imaging strategies, we were unable to analyze the pairwise comparison of all outcomes between different imaging strategies. Finally, there were only a few studies concerning on the rate of PGs inadvertent resection in ICGF, resulting in the lack of comparison between ICGF and other IVPG strategies in this aspect.

## Conclusion

Compared to NE, the three imaging strategies demonstrated significant superiority in the identification and functional protection of PGs during thyroid surgery. Each imaging strategy has its unique strengths and limitations. AF is the best strategy in reducing the incidence of postoperative hypocalcemia, increasing the rate of PG identification, and reducing the rate of PG inadvertent resection and auto-transplantation. However, AF fails to assess the blood perfusion of PGs. Although ICGF is less effective than AF in PG identification, it has great value in assessing PG viability, which leads to the trend towards PG auto-transplantation. CN is the best strategy in reducing the incidence of postoperative hypoparathyroidism, which is an ideal alternative in centres without AF and ICGF.

## Ethical approval

Not applicable.

## Consent

Not applicable.

## Source of funding

This work was supported by the Basic Research and Frontier Exploration Project of Yuzhong District, Chongqing, China (Grant No. 20210162), and the Chongqing Technology Innovation and Application Development Special Social Development Field Key Projects (Grant No. CSTB2022TIAD- KPX0177).

## Author contribution

D.L., B.P., and E.T. take responsibility for the integrity of the data and the accuracy of the data analysis. D.L., B.P., and E.T. contributed equally to this study and share first authorship. D.L., B.P., E.T.: conceptualization, data curation, formal analysis, methodology, writing—original draft. Y.S., Y.Y., T.Y., S.Y.: data curation, investigation, and funding acquisition. yang, zhang: funding acquisition, supervision, and writing—review and editing.

## Conflicts of interest disclosure

There are no conflicts of interest.

## Research registration unique identifying number (UIN)

This network meta-analysis was registered in the PROSPERO international database (NO. CRD42023452835, https://www.crd.york.ac.uk/prospero/).

## Guarantor

Zeyu Yang and Fan Zhang.

## Data statement

We declared that materials described in the manuscript, including all relevant raw data, will be freely available to any scientist wishing to use them for non-commercial purposes, without breaching participant confidentiality.

## Provenance and peer review

Not commissioned, externally peer-reviewed.

## Supplementary Material

SUPPLEMENTARY MATERIAL

## Supplementary Material

**Figure:**
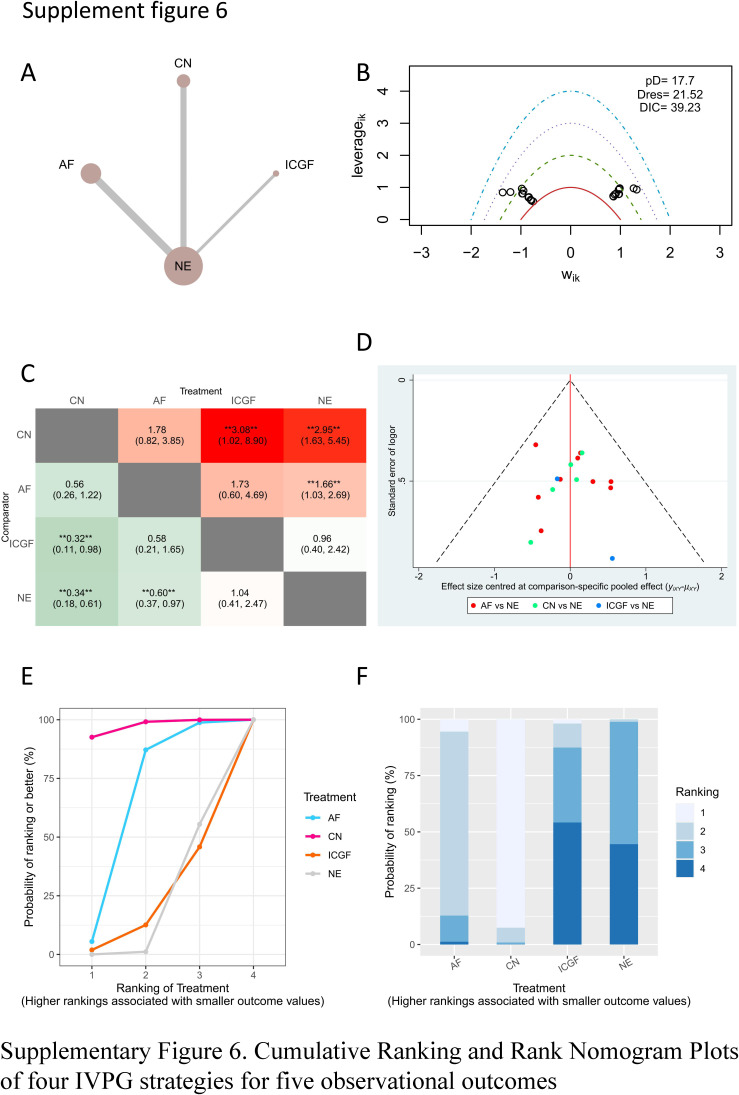


**Figure:**
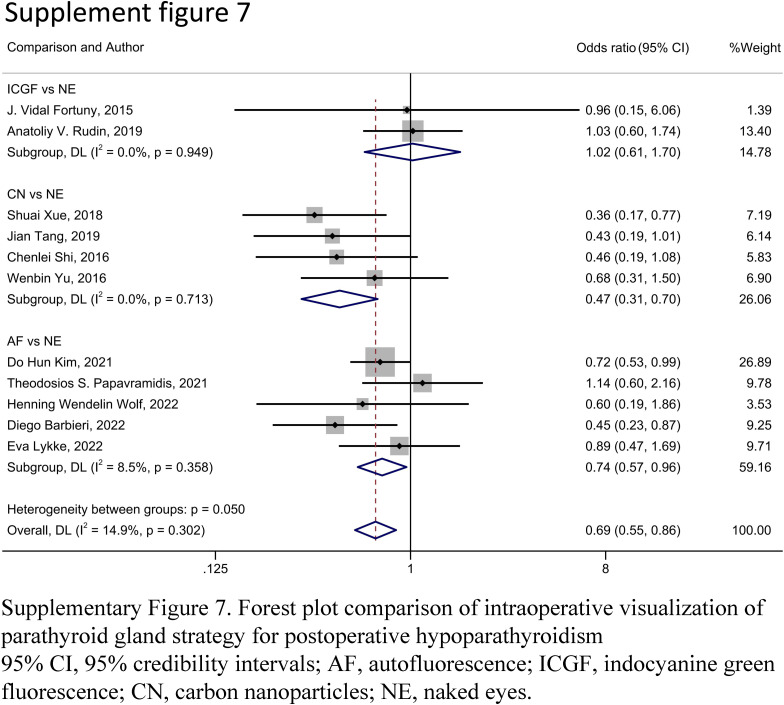


**Figure:**
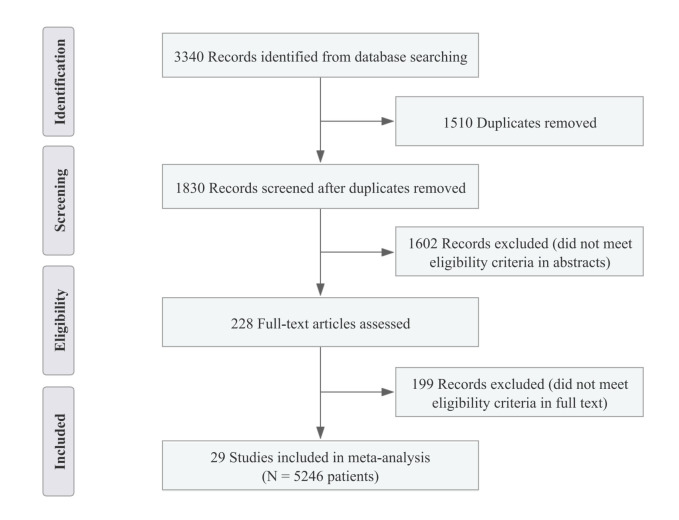


**Figure:**
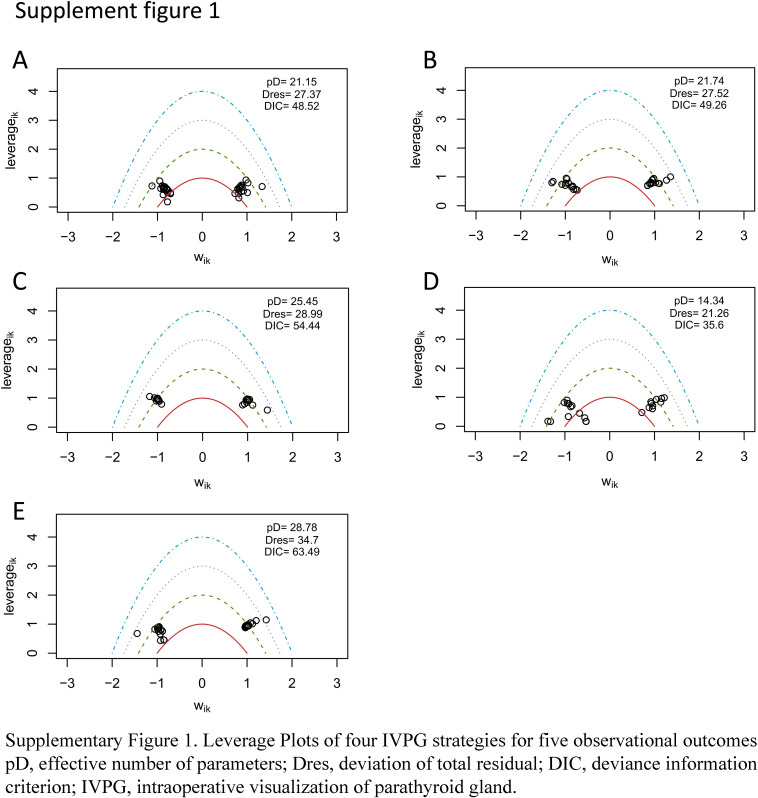


**Figure:**
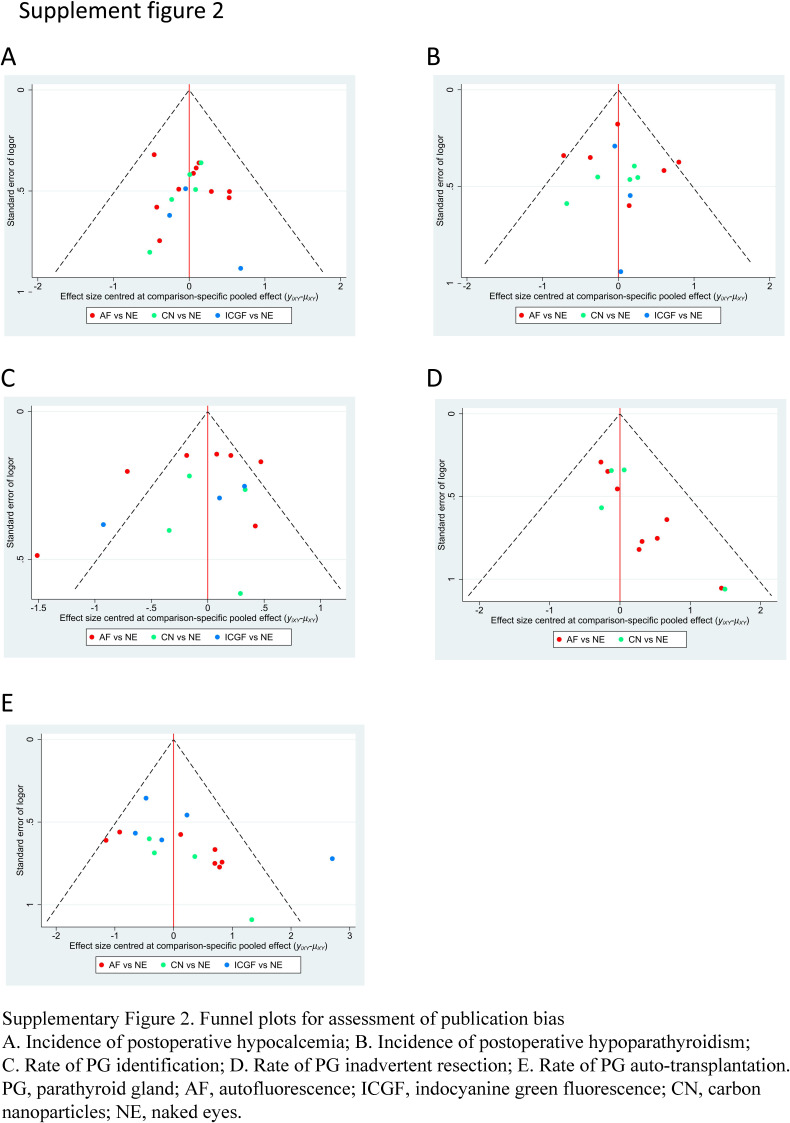


**Figure:**
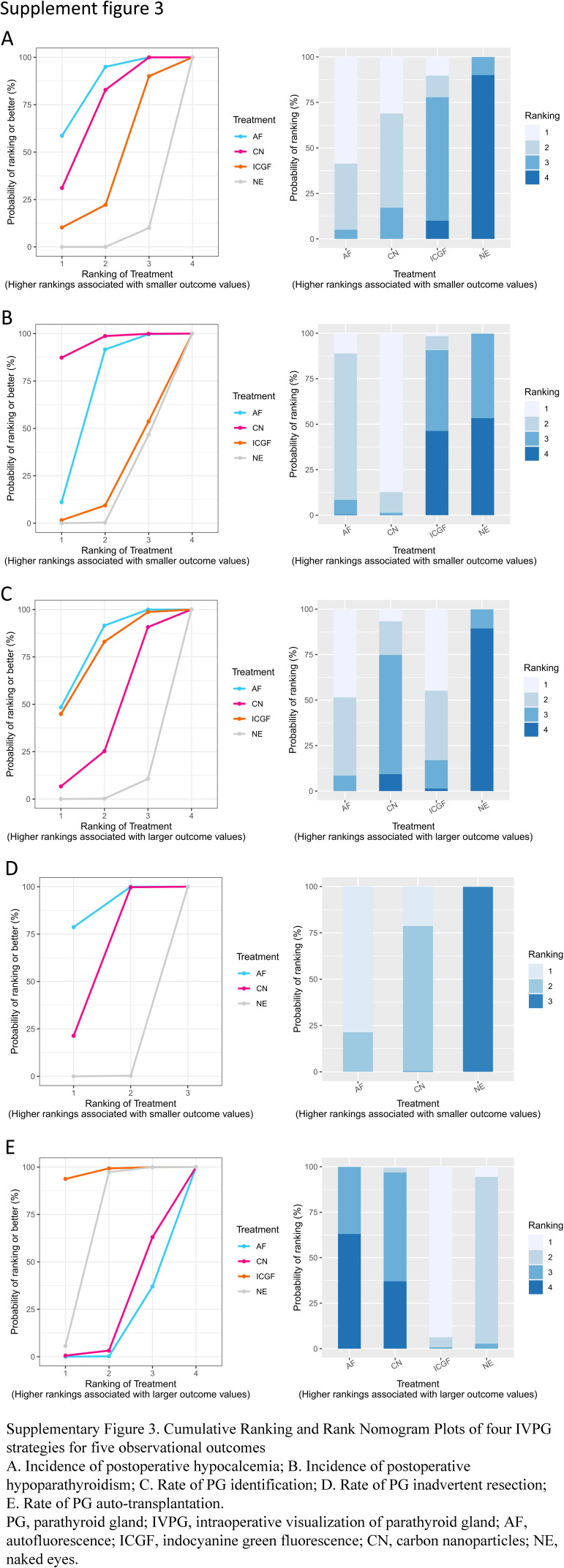


**Figure:**
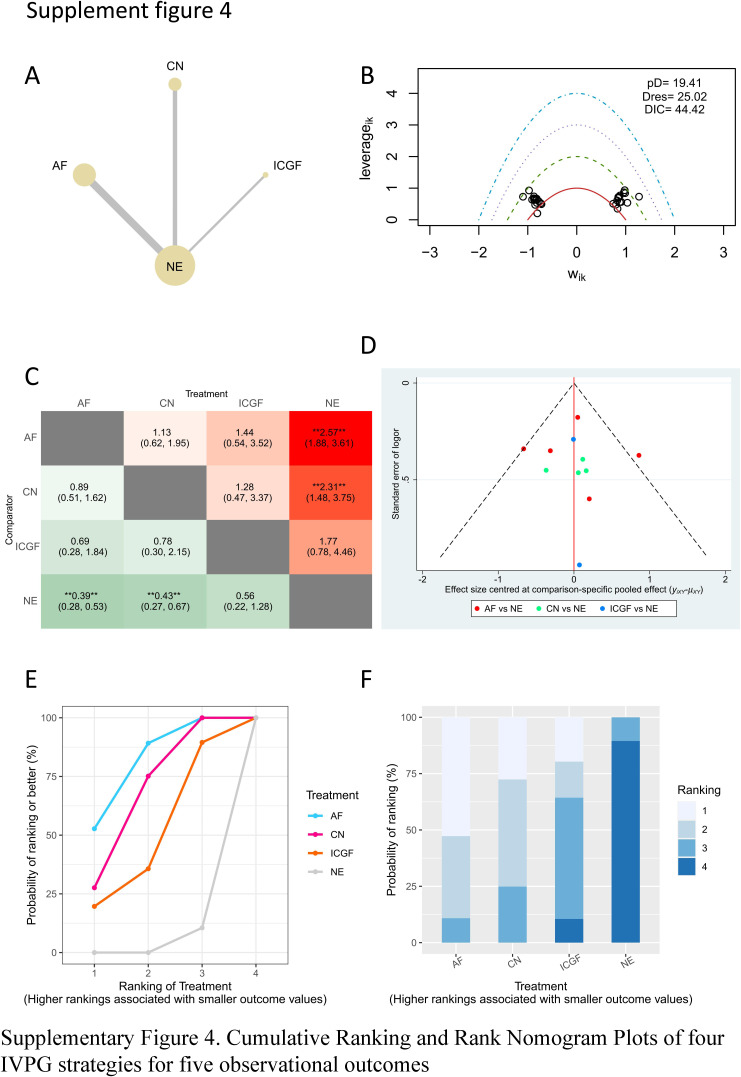


**Figure:**