# Supplementary material for: Intraoperative strategies in identification and functional protection of parathyroid glands for patients with thyroidectomy: a systematic review and network meta-analysis
Source: Int J Surg. 2023 Dec 11;110(3):1723–34. doi: 10.1097/JS9.0000000000000991 (PMC10942249; doi:10.1097/JS9.0000000000000991)
Supplement: SUPPLEMENTARY MATERIAL [file js9-110-1723-s006.pdf]

Supplement figure 1

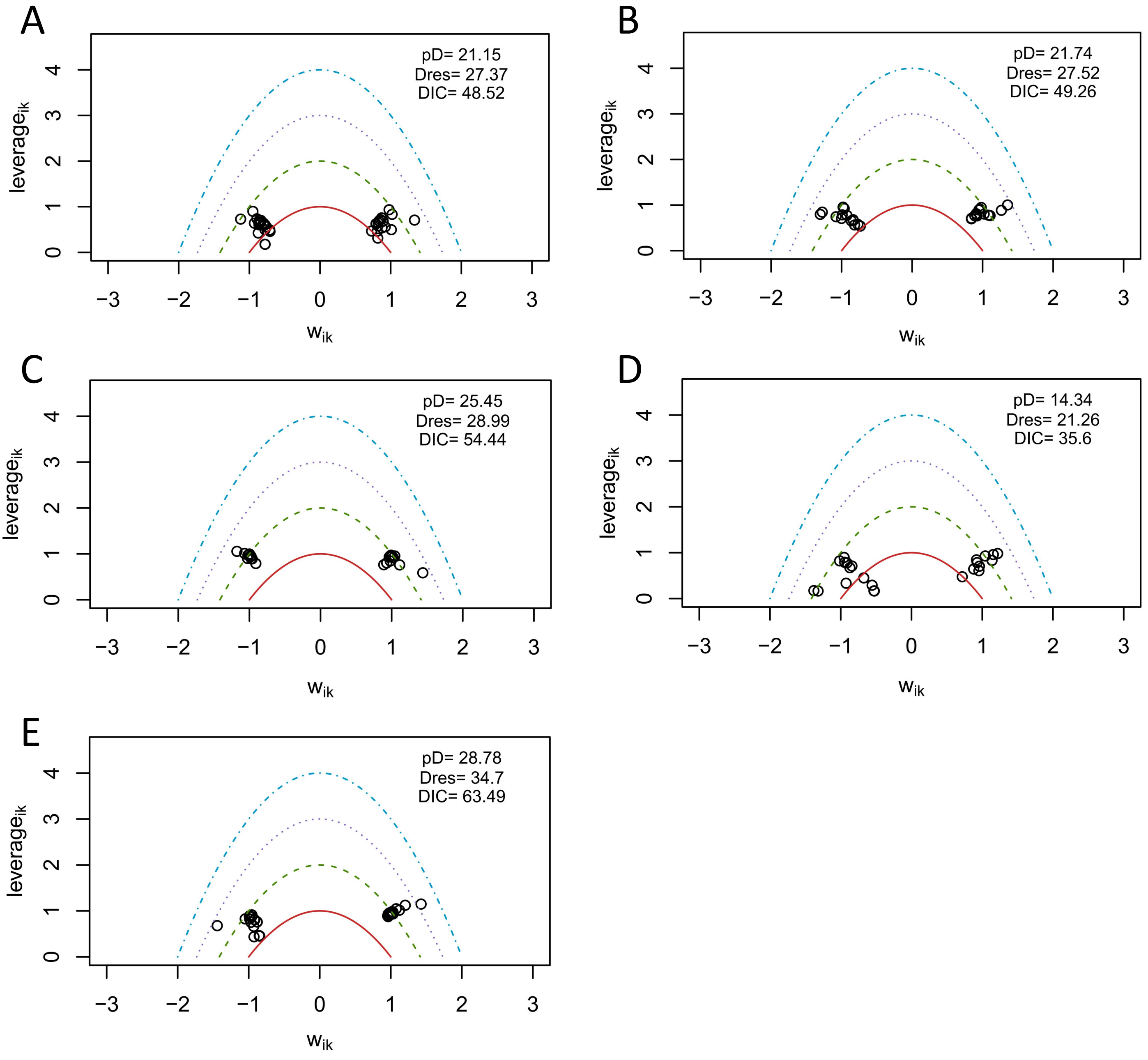

Supplement figure 2

A

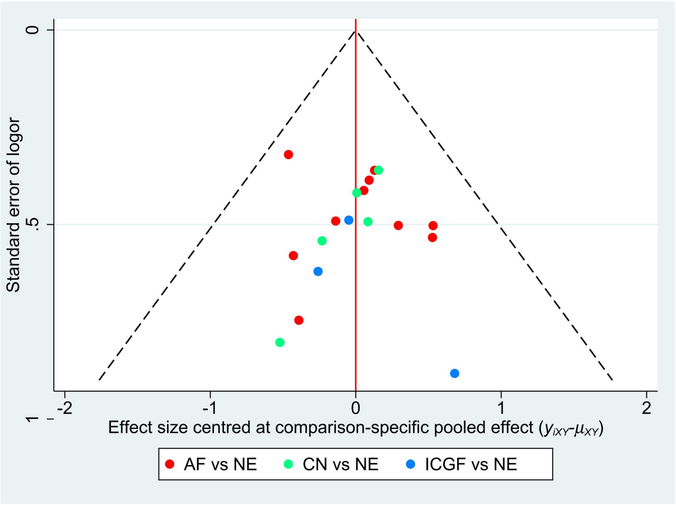

B

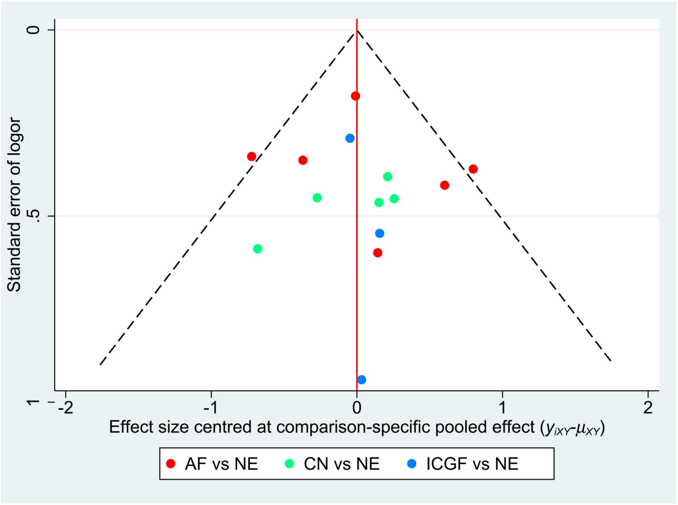

C

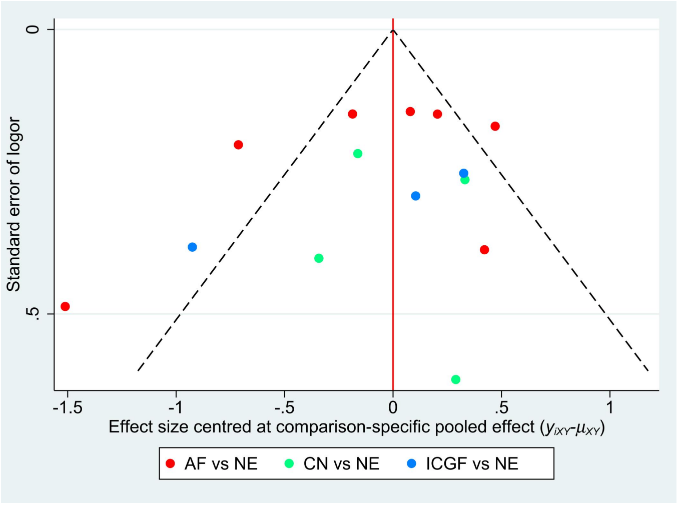

D

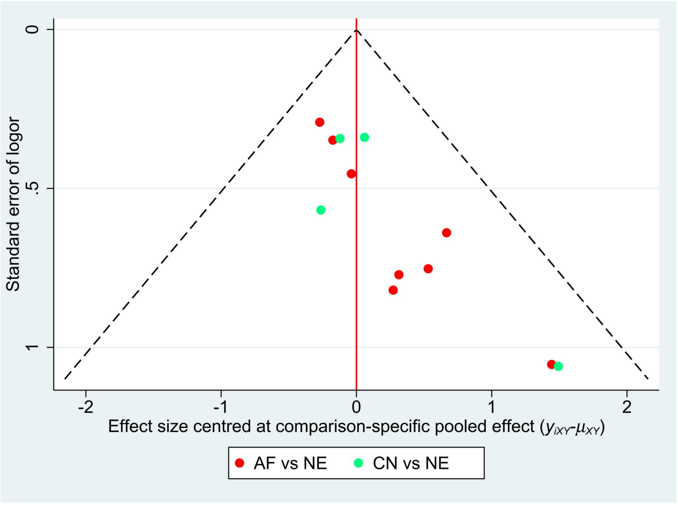

E

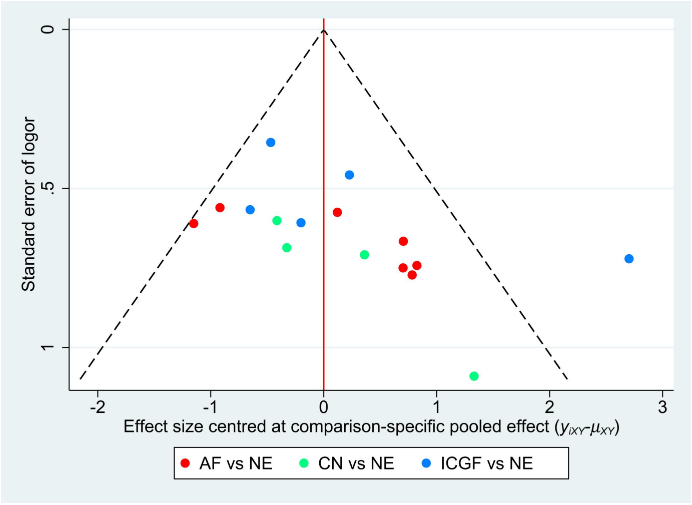

Supplement figure 3

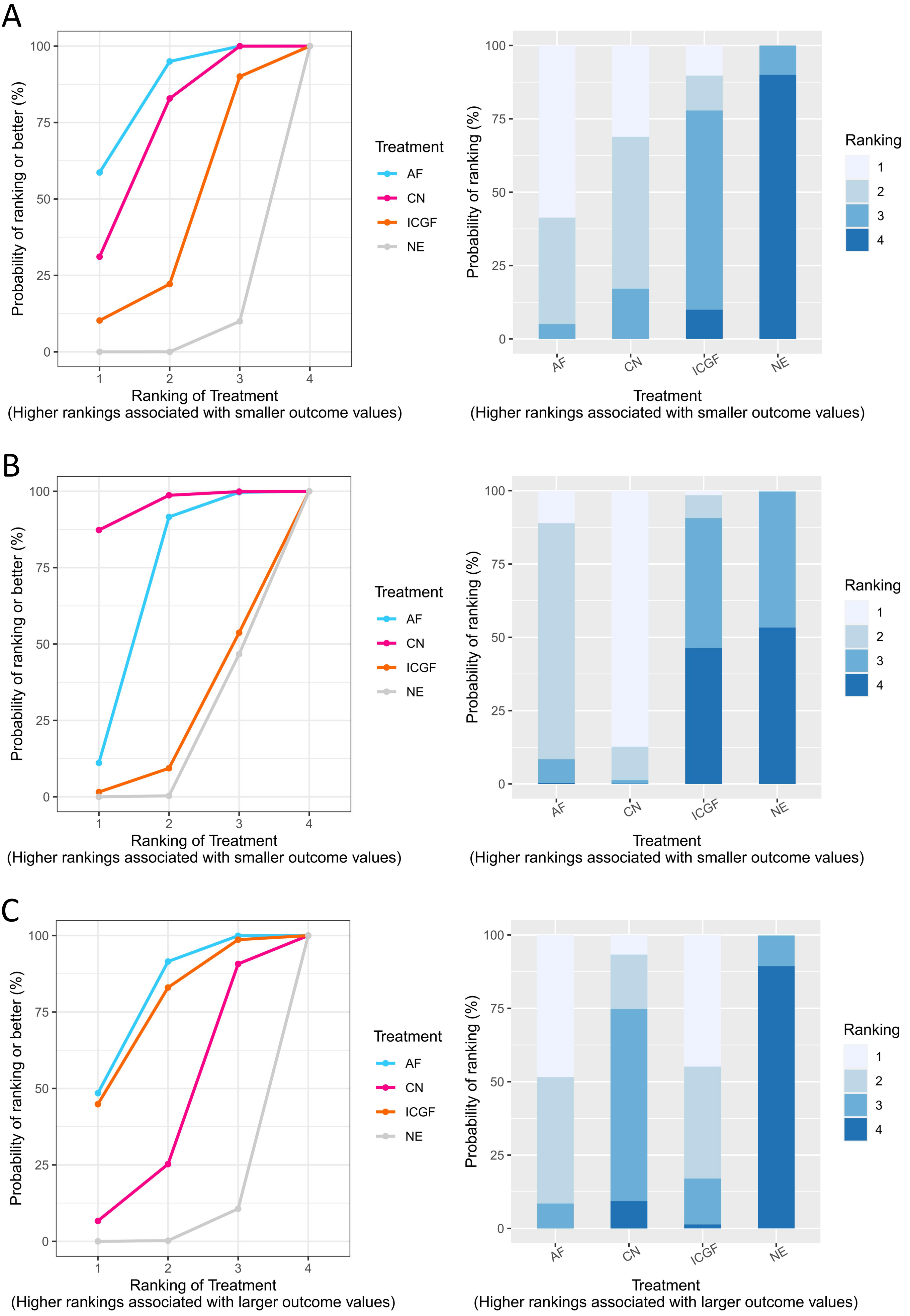

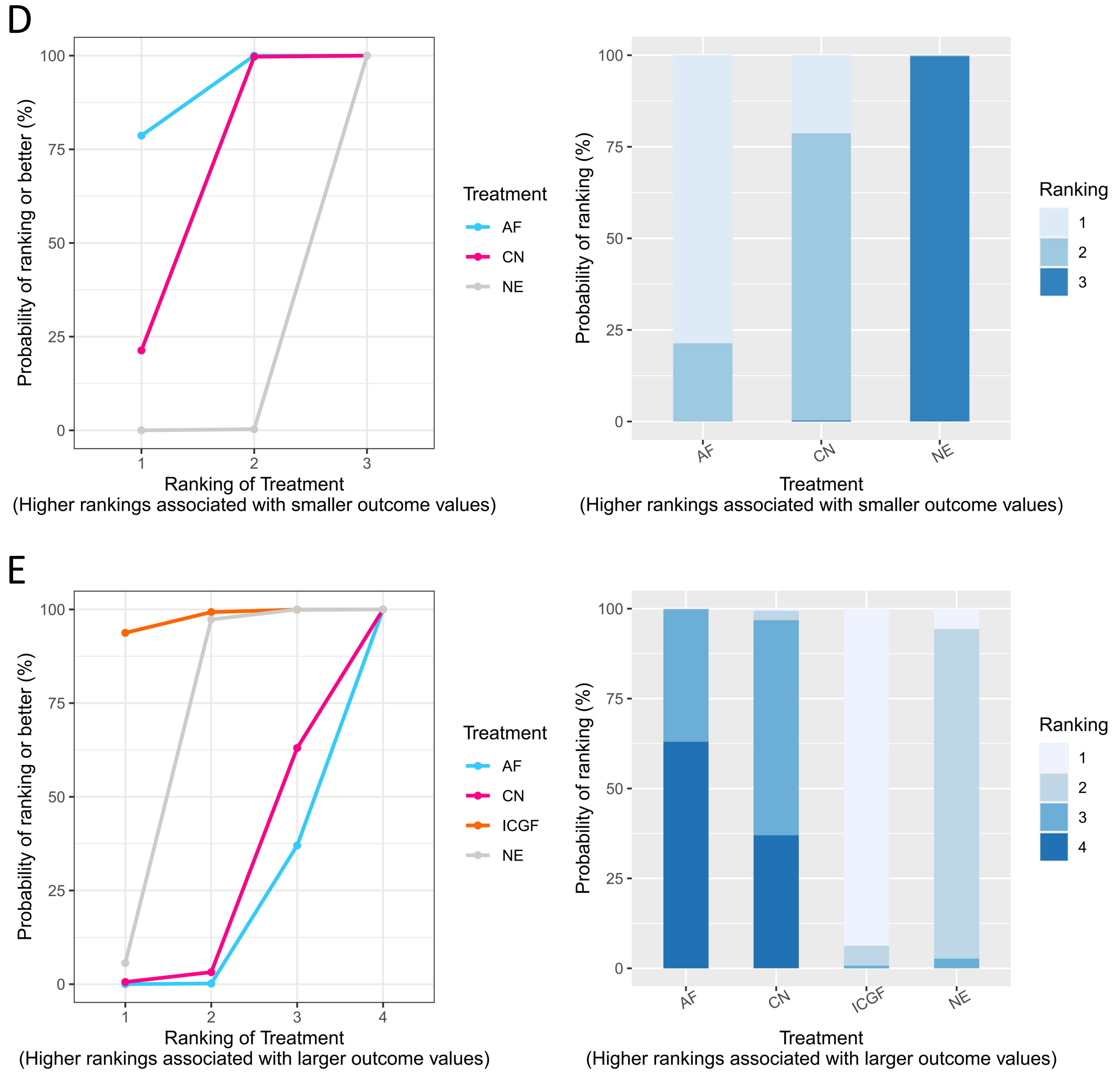
D
Probability of ranking or better (%)
Ranking of Treatment
(Higher rankings associated with smaller outcome values)
Treatment
AF
CN
NE
Probability of ranking (%)
Treatment
(Higher rankings associated with smaller outcome values)
Ranking
1
2
3
E
Probability of ranking or better (%)
Ranking of Treatment
(Higher rankings associated with larger outcome values)
Treatment
AF
CN
ICGF
NE
Probability of ranking (%)
Treatment
(Higher rankings associated with larger outcome values)
Ranking
1
2
3
4

Supplement figure 4

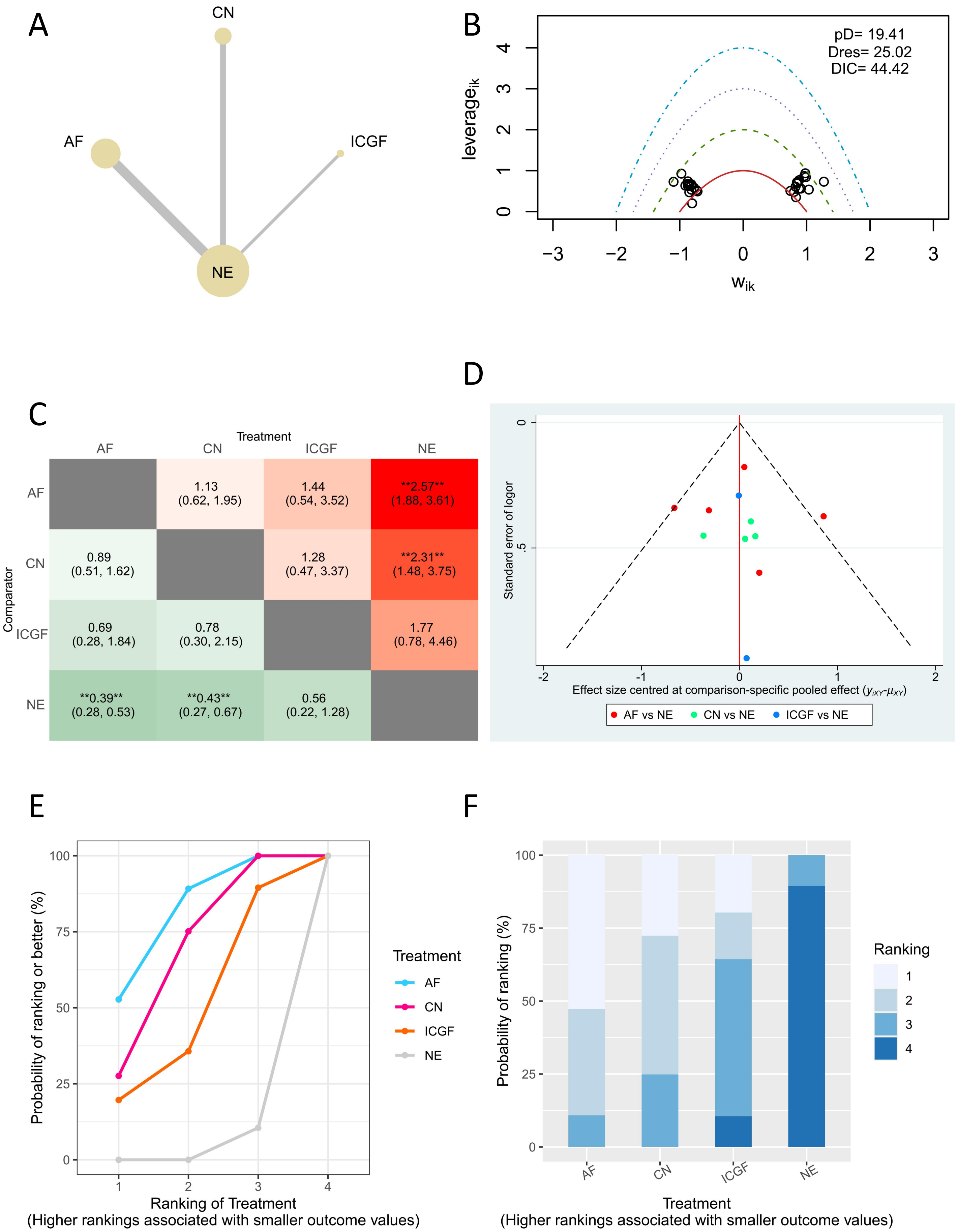

Supplement figure 5

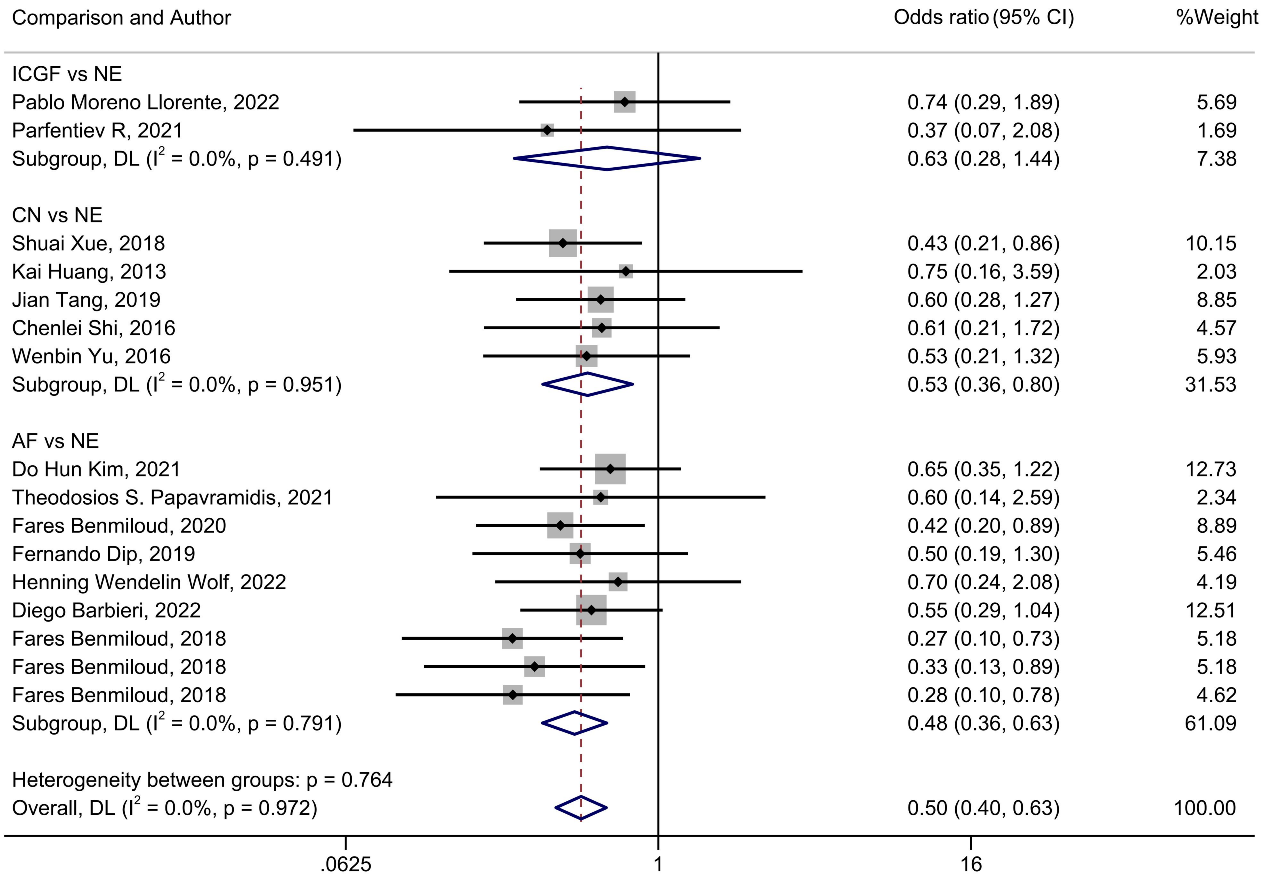

Supplement figure 6

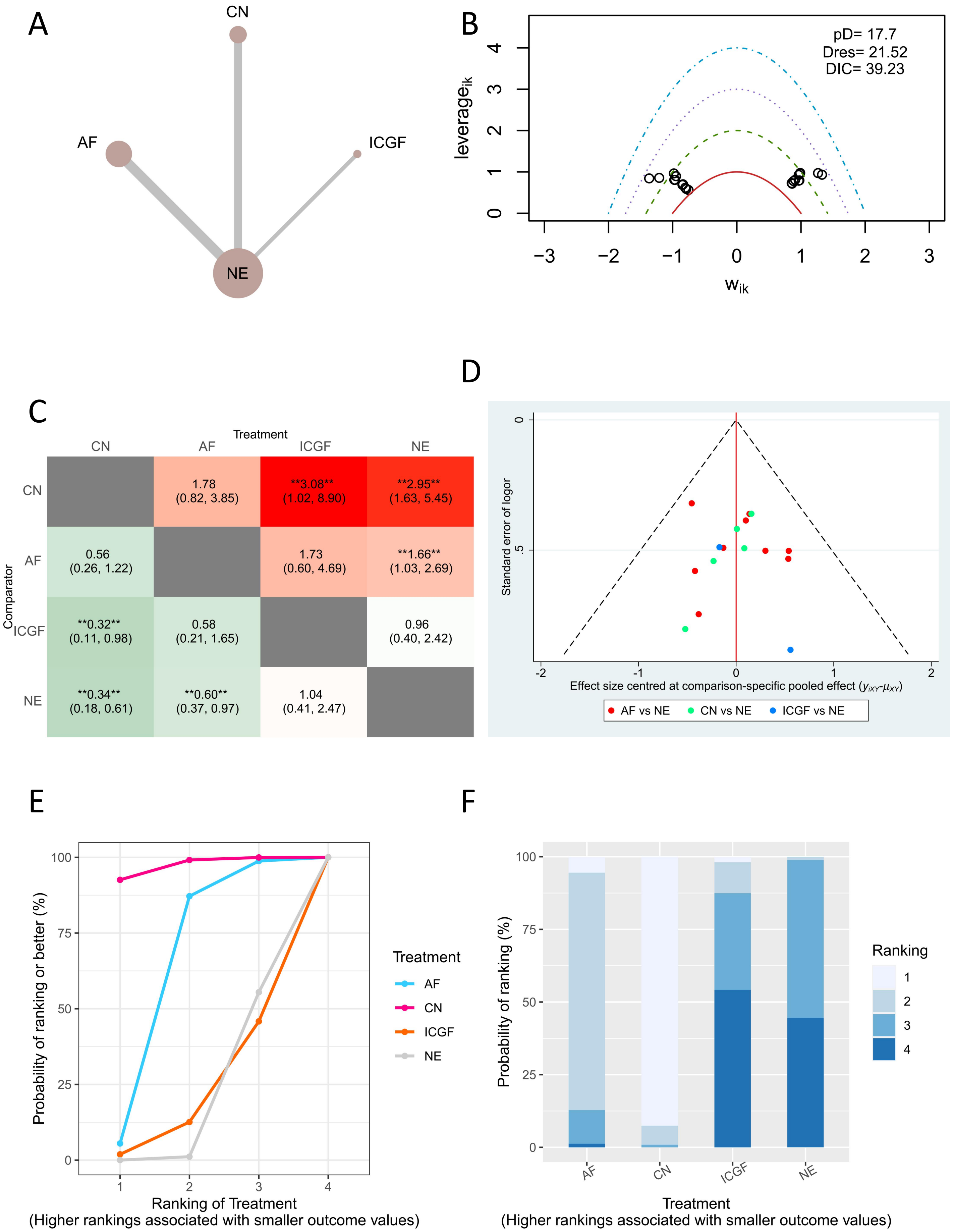

Supplement figure 7

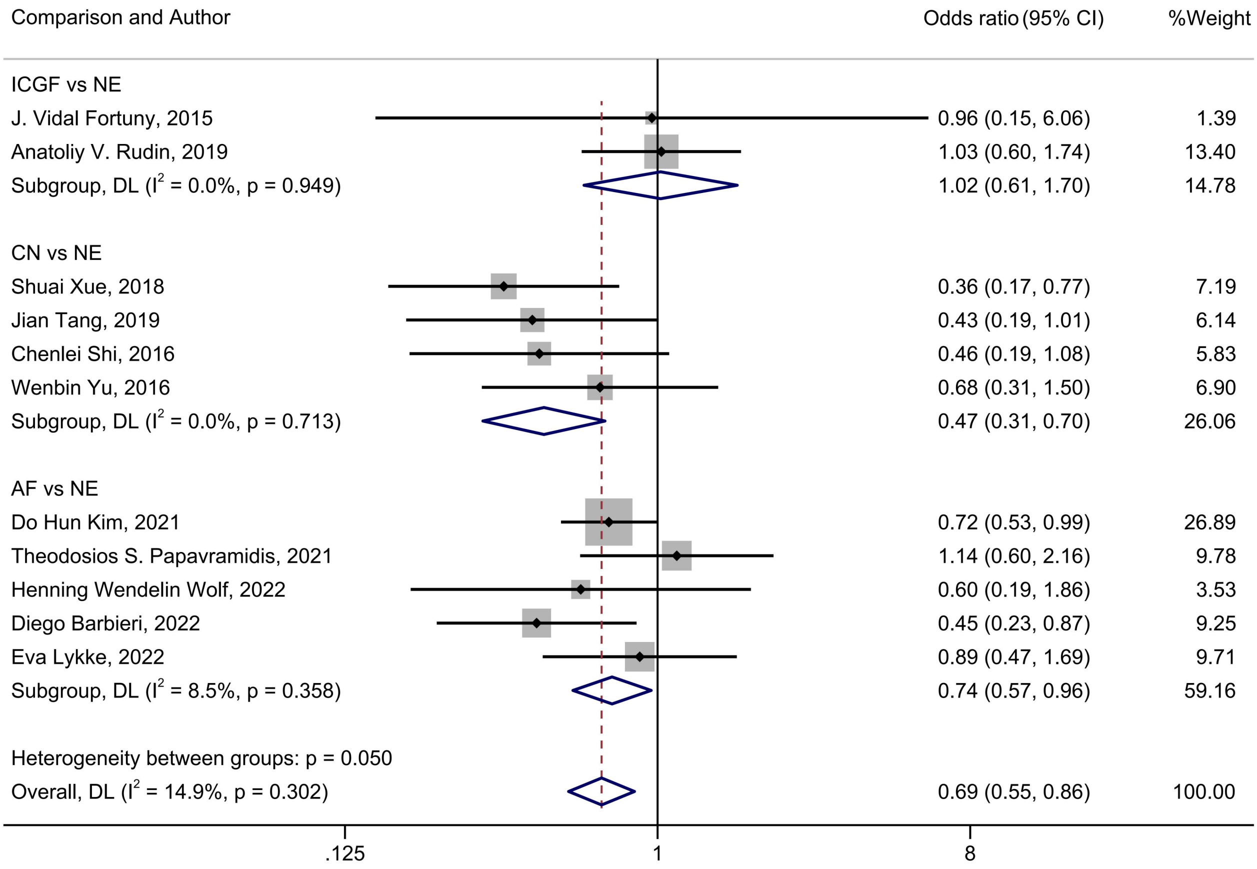

Supplementary Table 1. Search strategies of PubMed, Web of Science, Cochrane Central Register of Controlled Trials, Embase, Google Scholar database or CNKI.

**PubMed**			
**#ID**	**Topic or intervention**	**Query**	**Records**
#1	Thyroidectomy	"Thyroidectomy"[Mesh] OR Thyroidectomy[Title/Abstract] OR "thyroid surgery"[Title/Abstract] OR "thyroid operation"[Title/Abstract]	35 597
#2	Indocyanine green fluorescence	"Indocyanine green"[Mesh] OR “Green, Indocyanine”[Title/Abstract] OR Wofaverdi[Title/Abstract] OR Vophaverdin[Title/Abstract] OR Ujoveridin[Title/Abstract] OR Vofaverdin[Title/Abstract] OR “Cardio-Green”[Title/Abstract] OR “Cardio Green”[Title/Abstract] OR Cardiogreen[Title/Abstract]	9 907
#3	Autofluorescence	"Autofluorescence"[Mesh] OR Autofluorescence[Title/Abstract] OR “Imaging, Optical”[Title/Abstract] OR “Fluorescence Imaging”[Title/Abstract] OR “Imaging, Fluorescence”[Title/Abstract] OR “Fundus Autofluorescence Imaging”[Title/Abstract] OR “Autofluorescence Imaging, Fundus”[Title/Abstract] OR “Fundus Autofluorescence Imagings”[Title/Abstract] OR “Imaging, Fundus Autofluorescence”[Title/Abstract] OR “Autofluorescence Imaging”[Title/Abstract] OR “Imaging, Autofluorescence”[Title/Abstract]	29 713
#4	Near-infrared fluorescence	"near-infrared fluorescence"[Mesh] OR “infrared fluorescence”[Title/Abstract] OR “near-infrared fluorescence”[Title/Abstract] OR “near-infrared luminescence”[Title/Abstract] OR “infrared emission”[Title/Abstract] OR “infrared luminescence”[Title/Abstract]	4 805
#5	Carbon nanoparticles	"carbon nanoparticles"[Mesh] OR “carbon nanoparticles”[Title/Abstract] OR “nano-carbon”[Title/Abstract] OR “carbon nanoparticles”[Title/Abstract] OR “nanocrystalline carbon”[Title/Abstract] OR “nano-biochar”[Title/Abstract] OR “nano carbon particle”[Title/Abstract] OR “nano-carbonated”[Title/Abstract]	1 657
#6	All intervention	#2 OR #3 OR #4 OR #5	42 846
#7	Final query	#6 AND #1	**223**
**Web of Science**			
**#ID**	**Topic or intervention**	**Query**	**Records**
#1	Thyroidectomy	(TS=(Thyroidectomy) OR AB=（Thyroidectomy OR “thyroid surgery” OR “thyroid operation”)）	40 689
#2	Indocyanine green fluorescence	(TS=(“Indocyanine green”) OR AB=(“Indocyanine green” OR “Green, Indocyanine” OR Wofaverdin OR	22 953

		Vophaverdin OR Ujoveridin OR Vofaverdin OR “Cardio-Green” OR “Cardio Green” OR Cardiogreen))	
#3	Autofluorescence	(TS=(Autofluorescence) OR AB=(Autofluorescence OR “Imaging, Optical” OR “Fluorescence Imaging” OR “Imaging, Fluorescence” OR “Fundus Autofluorescence Imaging” OR “Autofluorescence Imaging, Fundus” OR “Fundus Autofluorescence Imagings” OR “Imaging, Fundus Autofluorescence” OR “Autofluorescence Imaging” OR “Imaging, Autofluorescence”))	38 474
#4	Near-infrared fluorescence	(TS=(“near-infrared fluorescence”) OR AB=("near-infrared fluorescence" OR “infrared fluorescence” OR “near-infrared fluorescence” OR “near-infrared luminescence” OR “infrared emission” OR “infrared luminescence”))	10 011
#5	Carbon nanoparticles	(TS=(“carbon nanoparticles”) OR AB=(“carbon nanoparticles” OR “nano-carbon” OR “nanocrystalline carbon” OR “nano-biochar” OR “nano carbon particle” OR “nano-carbonated”))	5 377
#6	All intervention	#2 OR #3 OR #4 OR #5	71 718
#7	Final query	#6 AND #1	**286**
**Cochrane Central Register of Controlled Trials**			
**#ID**	**Topic or intervention**	**Query**	**Records**
#1	Thyroidectomy	MeSH descriptor: [Thyroidectomy] explode all trees	811
#2		(Thyroidectomy OR “thyroid surgery” OR “thyroid operation”):ti,ab,kw	2 322
#3		#1 OR #2	2 322
#4	Indocyanine green fluorescence	MeSH descriptor: [Indocyanine Green] explode all trees	307
#5		(“Indocyanine green” OR “Green, Indocyanine” OR Wofaverdin OR Vophaverdin OR Ujoveridin OR Vofaverdin OR “Cardio-Green” OR “Cardio Green” OR Cardiogreen):ti,ab,kw	1 206
#6		#4 OR #5	1 206
#7	Autofluorescence	MeSH descriptor: [Optical Imaging] explode all trees	2 162
#8		(Autofluorescence OR “Imaging, Optical” OR “Fluorescence Imaging” OR “Imaging, Fluorescence” OR “Fundus Autofluorescence Imaging” OR “Autofluorescence Imaging, Fundus” OR “Fundus Autofluorescence Imagings” OR “Imaging, Fundus Autofluorescence” OR “Autofluorescence Imaging” OR “Imaging, Autofluorescence”):ti,ab,kw	797

#9		#7 OR #8	2 864
#10	Near-infrared fluorescence	MeSH descriptor: [Fluorescence] explode all trees	350
#11		(“near-infrared fluorescence” OR “infrared fluorescence” OR “near-infrared fluorescence” OR “near-infrared luminescence” OR “infrared emission” OR “infrared luminescence”):ti,ab,kw	105
#12		#10 OR #11	449
#13	Carbon nanoparticles	MeSH descriptor: [Carbon] explode all trees	1 347
#14		MeSH descriptor: [Nanoparticles] explode all trees	382
#15		#13 AND #14	43
#16		(“carbon nanoparticles” OR “nano-carbon” OR “nanocrystalline carbon” OR “nano-biochar” OR “nano carbon particle” OR “nano-carbonated”):ti,ab,kw	74
#17		#15 OR #16	90
#18	All intervention	#6 OR #9 OR #12 OR #17	4 242
#19	Final query	#3 AND #18	**63**
**Embase**			
**#ID**	**Topic or intervention**	**Query**	**Records**
#1	Thyroidectomy	thyroidectomy:ab,ti OR 'thyroid surgery':ab,ti OR 'thyroid operation':ab,ti	31 936
#2	Indocyanine green fluorescence	'indocyanine green':ab,ti OR 'green, indocyanine':ab,ti OR wofaverdin:ab,ti OR vophaverdin:ab,ti OR ujoveridin:ab,ti OR vofaverdin:ab,ti OR 'cardio-green':ab,ti OR 'cardio green':ab,ti OR cardiogreen:ab,ti	17 463
#3	Autofluorescence	'autofluorescence':ab,ti OR autofluorescence:ab,ti OR 'imaging, optical':ab,ti OR 'fluorescence imaging':ab,ti OR 'imaging, fluorescence':ab,ti OR 'fundus autofluorescence imaging':ab,ti OR 'autofluorescence imaging, fundus':ab,ti OR 'fundus autofluorescence imagings':ab,ti OR 'imaging, fundus autofluorescence':ab,ti OR 'autofluorescence imaging':ab,ti OR 'imaging, autofluorescence':ab,ti	27 861
#4	Near-infrared fluorescence	'infrared fluorescence':ab,ti OR 'near-infrared fluorescence':ab,ti OR 'near-infrared luminescence':ab,ti OR 'infrared emission':ab,ti OR 'infrared luminescence':ab,ti	4 574
#5	Carbon nanoparticles	'carbon nanoparticles':ab,ti OR 'nano-carbon':ab,ti OR 'nanocrystalline carbon':ab,ti OR 'nano-biochar':ab,ti OR 'nano carbon particle':ab,ti OR 'nano-carbonated':ab,ti	1 463

#6	All intervention	#2 OR #3 OR #4 OR #5	51 454
#7	Final query	#6 AND #1	**217**
**Google Scholar database or CNKI**			
**#ID**	**Database**	**Query**	**Records**
#1	Google Scholar database	(Thyroidectomy OR “thyroid surgery” OR “thyroid operation”) AND ((“Indocyanine green” OR “Green, Indocyanine” OR Wofaverdin OR Vophaverdin OR Ujoveridin OR Vofaverdin OR “Cardio-Green” OR “Cardio Green” OR Cardiogreen) OR (Autofluorescence OR “Imaging, Optical” OR “Fluorescence Imaging” OR “Imaging, Fluorescence” OR “Fundus Autofluorescence Imaging” OR “Autofluorescence Imaging, Fundus” OR “Fundus Autofluorescence Imagings” OR “Imaging, Fundus Autofluorescence” OR “Autofluorescence Imaging” OR “Imaging, Autofluorescence”) OR (“near-infrared fluorescence” OR “infrared fluorescence” OR “near-infrared fluorescence” OR “near-infrared luminescence” OR “infrared emission” OR “infrared luminescence”) OR (“carbon nanoparticles” OR “nano-carbon” OR “nanocrystalline carbon” OR “nano-biochar” OR “nano carbon particle” OR “nano-carbonated”))	**2 480**
#2	CNKI	SU%='甲状腺'*'手术' AND SU%='自体荧光' +'吲哚菁绿' +'纳米碳'	**71**

Supplementary Table 2. Characteristics of the 29 studies

Source	Intervention	No. of Patients	Female (%)	Age, y[2]	Surgical procedure	Patients of auto-transplantation	PGs of identification (%)	PGs of inadvertent resection (%)	Patients of Hypocalcemia	Patients of Hypoparathyroidism
Hui Ouyang, 2022	ICGF	34	28 (82.4)	34.4 (8.6)	Robotic	19	127 (93.4)	-	5	7
	NE	47	37 (78.7)	35.4 (9.4)	Robotic	18	138 (73.4)	-	8	14
Pablo Moreno Llorente, 2023	ICGF	36	25 (69.4)	48.0 (14.1)	Open	-	127 (88.2)	-	7	-
	NE	84	58 (69.0)	52.8 (13.5)	Open	-	270 (80.4)	-	22	-
Parfentiev R, 2021	ICGF	30	16 (53.3)	44.4 (11.2)	Open	11	-	-	2	-
	NE	28	18 (64.3)	43.2 (12.1)	Open	4	-	-	5	-
J. Vidal Fortuny, 2015	ICGF	36	-	-	Open	9	-	-	-	2
	NE	52	-	-	Open	5	-	-	-	3
Anatoliy V. Rudin, 2019	ICGF	86	69 (80.2)	47 (-)	Open	31	-	-	-	32
	NE	124	92 (74.2)	49 (-)	Open	15	-	-	-	45
J. Quéré, 2022	ICGF	32	26 (81.3)	54 (-)	Open	-	77 (60.2)	-	-	-
	NE	32	26 (81.3)	54 (-)	Open	-	65 (50.8)	-	-	-
Volodymyr V. Grubnik, 2023	ICGF	44	32 (72.7)	47.2 (6.4)	Open	16	-	-	-	-
	NE	48	33 (68.8)	44.6 (5.6)	Open	5	-	-	-	-
Shuai Xue, 2018	CN	106	86 (81.1)	44.5 (7.8)	Open	-	-	-	10	8
	NE	300	228 (76.0)	44.4 (14.9)	Open	-	-	-	66	63
Kai Huang,	CN	36	24 (66.7)	41.2 (2.5)	Open	-	-	-	3	-

2013	NE	36	26 (72.6)	40.1 (2.4)	Open	-	-	-	4	-
Jian Tang, 2019	CN	50	38 (76.0)	47.2 (11.8)	Open	-	-	-	15	10
	NE	50	40 (80.0)	48.1 (9.6)	Open	-	-	-	25	23
Chenlei Shi, 2016	CN	52	46 (88.5)	-	Open	1	152 (73.1)	-	7	10
	NE	45	39 (86.7)	-	Open	7	108 (60.0)	-	10	19
Wenbin Yu, 2016	CN	41	33 (80.5)	41.6 (17.1)	Open	3	-	-	9	15
	NE	41	30 (73.2)	41.7 (18.9)	Open	9	-	-	17	22
Daqi Zhang, 2020	CN	43	39 (90.7)	32.7 (6.3)	Endo	-	167 (97.1)	1 (0.6)	-	6
	NE	45	40 (88.9)	31.8 (7.5)	Endo	-	174 (96.7)	9 (5.0)	-	8
Lei Min, 2020	CN	120	92 (76.7)	40.1 (12.4)	Endo	4	-	16 (3.3)	-	-
	NE	86	41 (47.7)	39.6 (11.2)	Endo	5	-	20 (5.8)	-	-
Jie He, 2021	CN	54	52 (96.3)	34.7 (9.8)	Endo	-	106 (76.8)	-	-	-
	NE	72	70 (97.2)	35.0 (9.1)	Endo	-	138 (75.0)	-	-	-
Zhiliang Xu, 2019	CN	38	35 (92.1)	30.5 (7.0)	Endo	6	63 (82.9)	6 (7.9)	-	-
	NE	34	31 (91.2)	32.6 (7.2)	Endo	8	47 (69.1)	8 (11.8)	-	-
Tianhao Xie, 2022	CN	260	206 (79.2)	42.4 (13.3)	Open	-	-	14 (2.3)	-	-
	NE	245	198 (80.8)	43.9 (10.2)	Open	-	-	25 (4.8)	-	-
Do Hun Kim, 2021	AF	120	92 (76.7)	40.1 (12.4)	Open	-	-	18 (1.7)	17	88
	NE	86	41 (47.7)	39.6 (11.2)	Open	-	-	36 (3.2)	28	131

Theodosios S. Papavramidis, 2021	AF	261	206 (78.9)	51.3 (12.4)	Open	-	-	13 (3.6)	3	25
	NE	281	227 (80.8)	52.8 (10.9)	Open	-	-	26 (7.2)	5	22
Fares Benmiloud, 2020	AF	90	63 (70.0)	48.4 (14.6)	Open	4	390 (80.6)	3 (0.6)	11	-
	NE	90	70 (77.8)	45.7 (13.6)	Open	16	299 (62.3)	14 (2.9)	26	-
Fernando Dip, 2019	AF	121	96 (79.3)	52.5 (8.5)	Open	-	300 (88.2)	-	7	-
	NE	120	95 (79.2)	51.0 (9.5)	Open	-	217 (63.8)	-	14	-
Henning Wendelin Wolf, 2022	AF	85	67 (78.8)	48.7 (13.5)	Open	8	-	-	7	6
	NE	85	59 (69.4)	45.8 (13.7)	Open	11	-	-	10	10
Junwei Huang, 2023	AF	30	21 (70.0)	57.0 (12.7)	Endo	3	195 (97.5)	1 (0.5)	18	17
	NE	30	22 (73.0)	59.0 (11.9)	Endo	17	161 (80.5)	10 (5.0)	30	31
Diego Barbieri, 2022	AF	50	37 (74.0)	47.88 (-)	Open	-		-	21	17
	NE	50	37 (74.0)	43.03 (-)	Open	-	-	-	38	38
Fares Benmiloud, 2018	AF	93	75 (80.6)	49.6 (14.5)	Open	2	284 (76.3)	2 (0.5)	5	-
	NE	153	116 (75.8)	50.1 (13.9)	Open	23	401 (65.5)	11 (1.8)	30	-
	NE	180	140 (77.8)	53.8 (14.1)	Open	30	451 (62.6)	16 (2.2)	29	-
	NE	87	67 (77.0)	54.3 (13.2)	Open	14	248 (76.3)	6 (1.7)	17	-
Yoo Seok Kim, 2020	AF	100	81 (81.0)	51.6 (15.2)	Open	-	-	6 (1.5)	-	-
	NE	200	160 (80.0)	50.2 (15.5)	Open	-	-	28 (3.5)	-	-
A DiMarco,	AF	106	88 (83.0)	48.0 (14.2)	Open	-	-	-	5	-

2019	NE	163	133 (81.6)	47.9 (15.8)	Open	-	-	-	9	-
Eva Lykke, 2022	AF	69	50 (72.5)	57.4 (16)	Open	5	--	-	-	22
	NE	78	60 (76.9)	52.2 (15.8)	Open	7	-	-	-	28
M. Pastoricchio, 2022	AF	25	17 (68.0)	52 (-)	Open	-	68 (81.9)	-	-	-
	NE	26	19 (73.1)	55.5 (-)	Open	-	63 (76.8)	-	-	-

PG, parathyroid gland; AF, autofluorescence; CN, carbon nanoparticles; ICGF, indocyanine green fluorescence; NE, naked eye.

Supplementary Table 3. References quality evaluation by Newcastle-Ottawa Scale

Study	SELECTION				COMPARABILITY	EXPOSURE			
	Adequate Definition of Cases	Representati -veness of the Cases	Selection of Controls	Definition of Controls	Comparability of Cases and Controls	Ascertainment of Exposure	Same Method of ascertainment for Cases and Controls	Non-Response Rate	Total scores
A DiMarco 2019	★	★	★	★	★★	★	★	★	9
Anatolia V. Rubin 2019	★	★	★	★	★★	★	★	★	9
Chenlei Shi 2016	★	★	★	★	★★	★	★	★	9
Do Hun Kim 2021	★	★	★	★	★★	★	-	★	8
Fares Benmiloud 2018	★	★	★	★	-	★	★	★	7
Hui Ouyang 2022	★	★	★	★	★★	★	★	-	8
J.Quere 2022	★	★	★	★	★	★	★	-	7
Jian Tang 2019	-	-	★	★	★★	★	★	-	6
Tianhao Xie 2021	★	-	★	★	★★	★	★	-	7
M.Pastoricchio 2022	★	★	★	★	★★	★	★	-	8
Theodosios S.P 2021	★	★	★	★	★★	★	★	-	8
Yoo Seok Kim 2020	★	-	★	★	★★	★	★	★	8
Zhiliang Xu 2019	★	-	★	★	★★	★	★	-	7

J. Vidal Fortuny 2015	★	-	★	★	★	★	★	-	6
Volodymyr V. Grubnik, 2023	★	★	★	★	★★	★	★	-	8
Shuai Xue 2018	★	-	★	★	★★	★	★	-	7
Lei Min 2020	★	-	★	★	★	★	★	★	6
Pablo Moreno Llorente 2023	★	-	★	★	★★	★	★	★	8
Junwei Huang 2023	★	-	★	★	★★	★	★	-	7
Diego Barbieri 2022	★	★	★	★	★★	★	★	-	8
Jie He 2021	★	★	★	★	★	★	★	★	8

Supplementary Table 4. References quality evaluation by Cochrane Collaboration tool.

Study	Randomization process	Deviations from intended interventions	Mising outcome data	Measurement of the outcome	Selection of the reported result	Overall Bias
Daqi Zhang 2020	Some concerns	Low	Low	Low	Low	Some concerns
Fares Benmiloud 2020	Low	Low	Low	Low	Low	Low
Fernando Dip 2019	Some concerns	Low	Low	Low	Some concerns	Some concerns
Parfentiev R 2021	Some concerns	Low	Some concerns	Low	Low	Some concerns
Wenbin Yu 2016	Some concerns	Low	Low	Low	Low	Some concerns
Kai Huang 2013	Low	Some concerns	High	Low	Some concerns	High
Eva Lykke 2022	Low	Low	Low	Low	Low	Low
Henning Wendelin Wolf 2022	Low	Low	Low	Low	Low	Low

Supplementary Table 5. The SUCRA values of all outcomes

Group	Postoperative hypocalcemia	Postoperative Hypoparathyroidism	Rate of PG identification	Rate of PG inadvertent resection	Rate of PG auto-transplantation
AF	0.85	0.67	0.80	0.89	0.12
CN	0.71	0.95	0.41	0.61	0.22
ICGF	0.41	0.22	0.76	-	0.98
NE	0.03	0.16	0.04	0	0.68

SUCRA, the surface under the cumulative ranking curve; PG, parathyroid gland; AF, autofluorescence; CN, carbon nanoparticles; ICGF, indocyanine green fluorescence; NE, naked eye.

Supplementary Table 6. The SUCRA values of all outcomes in the traditional open thyroid surgery group

	AF	CN	ICGF	NE
Postoperative hypocalcemia	0.79	0.68	0.50	0.03
Postoperative Hypoparathyroidism	0.64	0.97	0.20	0.19

SUCRA, the surface under the cumulative ranking curve; AF, autofluorescence; CN, carbon nanoparticles; ICGF, indocyanine green fluorescence; NE, naked eye.
